# An *in vitro* quantitative systems pharmacology approach for deconvolving mechanisms of drug-induced, multilineage cytopenias

**DOI:** 10.1371/journal.pcbi.1007620

**Published:** 2020-07-23

**Authors:** Jennifer L. Wilson, Dan Lu, Nick Corr, Aaron Fullerton, James Lu

**Affiliations:** 1 Department of Clinical Pharmacology, Genentech, Inc., South San Francisco, California, United States of America; 2 Department of Bioengineering and Therapeutic Sciences, University of California San Francisco, San Francisco, California, United States of America; 3 Department of Safety Assessment, Genentech, Inc., South San Francisco, California, United States of America; University at Buffalo - The State University of New York, UNITED STATES

## Abstract

Myelosuppression is one of the most common and severe adverse events associated with anti-cancer therapies and can be a source of drug attrition. Current mathematical modeling methods for assessing cytopenia risk rely on indirect measurements of drug effects and primarily focus on single lineage responses to drugs. However, anti-cancer therapies have diverse mechanisms with varying degrees of effect across hematopoietic lineages. To improve predictive understanding of drug-induced myelosuppression, we developed a quantitative systems pharmacology (QSP) model of hematopoiesis *in vitro* for quantifying the effects of anti-cancer agents on multiple hematopoietic cell lineages. We calibrated the system parameters of the model to cell kinetics data without treatment and then validated the model by showing that the inferred mechanisms of anti-proliferation and/or cell-killing are consistent with the published mechanisms for three classes of drugs with different mechanisms of action. Using a set of compounds as a reference set, we then analyzed novel compounds to predict their mechanisms and magnitude of myelosuppression. Further, these quantitative mechanisms are valuable for the development of translational *in vivo* models to predict clinical cytopenia effects.

## Author summary

Reduced bone marrow activity and levels of mature blood cells is an undesirable side effect of many anti-cancer therapies. Selecting promising lead compounds for further development requires understanding of potential myelosuppressive effects. However, existing preclinical experiments and modeling formulations fail to consider drug effects on multiple blood cell types or the mechanistic differences between how drugs induced myelosuppression. Here we developed a quantitative systems pharmacology (QSP) model that estimates a drug candidate’s effect on multiple precursor and mature blood cell lineages and further distinguishes how the drug affects these populations—through cell-killing or anti-proliferation mechanisms. This modeling formalism is valuable for vetting compounds for therapeutic development and for further translational modeling to anticipate the clinical effects of compounds.

This is a *PLOS Computational Biology* Methods paper.

## Introduction

Drug-induced myelosuppression is one of the most severe adverse events (AEs) associated with anti-cancer therapies[1]. Myelosuppression increases patient fatigue and hinders their daily routines[2,3], and increases patient risk for infection [4]. Understanding patient propensity for AEs is required for clinical optimization of both drug selection and dose schedules. Often anti-cancer therapies specifically optimize efficacy on the basis of minimizing undesirable myelosuppressive effects [5,6]. Despite the frequency of myelosuppression following anti-cancer treatment, predicting the severity of this AE remains challenging[1].

Computational models and pre-clinical experiments remain the standard for anticipating and understanding potential myelosuppressive effects. Predictive toxicology approaches can expedite early phase clinical trials and reduce the number of patients treated with ineffective doses[7]. A validation study of 20 compounds demonstrated that pre-clinical *in vitro* measurements of a drug’s 90% inhibition concentrations (IC90) of granulocyte-macrophages was a sufficient predictor of the maximum tolerated dose (MTD) in animals and humans[7]. Many modeling approaches have captured the effects of novel compounds on single lineages. For instance, the Friberg model describes the *in vivo* development of neutrophils using multiple transit compartments where drug treatment can affect the self-renewal and proliferation of immature cell types[8]. Importantly, these models have supported safety-mitigating strategies in the clinic. Semi-mechanistic modeling combined with clinical data sufficiently captured G-CSF response and neutrophil loss after chemotherapy[9] and identified an optimal blood monitoring schedule during palbociclib treatment[10].

An understanding of mechanistic and lineage-specific effects would advance predictive toxicology approaches. Improved understanding of drug-induced myelosuppression requires a systems-level perspective of hematopoiesis and effects on progenitors to better explain downstream effects on blood cells[11]. A challenge to mathematical modeling of myelosuppression is understanding lineage effects in the bone marrow, especially when using indirect measurements in peripheral blood[3,11,12], suggesting *in vitro* measurements will be essential to this advancement. A cell-based assay that analyzed the relative anti-proliferative effects of multiple chemotherapies found that the extent of anti-proliferation was associated with the severity of myelosuppression[13]. These findings further suggest that a mechanistic understanding of drug-induced cytopenias can inform vetting of multiple drug candidates.

Modeling effects on multiple lineages and progenitors could be valuable for interpreting differences in toxicity induced by multiple compounds[3,11], yet advancing predictability requires better mechanistic understanding. For instance, a decrease in neutrophils could be a result of depletion of mature neutrophils or a depletion of granulocyte progenitors. One recent study used rat to human translation to understand how carboplatin-induced DNA damage affected multiple hematopoietic lineages[12]. A key feature of their approach was using QSP modeling to learn carboplatin effects on early hematopoietic progenitors in rats and applying this mechanistic understanding to predict clinical rates of cytopenias. They discovered that feedback on multipotent progenitor (MPP) proliferation was insufficient for capturing clinical recoveries, but that adding feedback on MPP maturation could adequately describe clinical data[12]. This demonstrates that a mechanistic understanding of cytopenias is valuable for creating meaningful, translational *in vivo* models.

**Fig 1:**
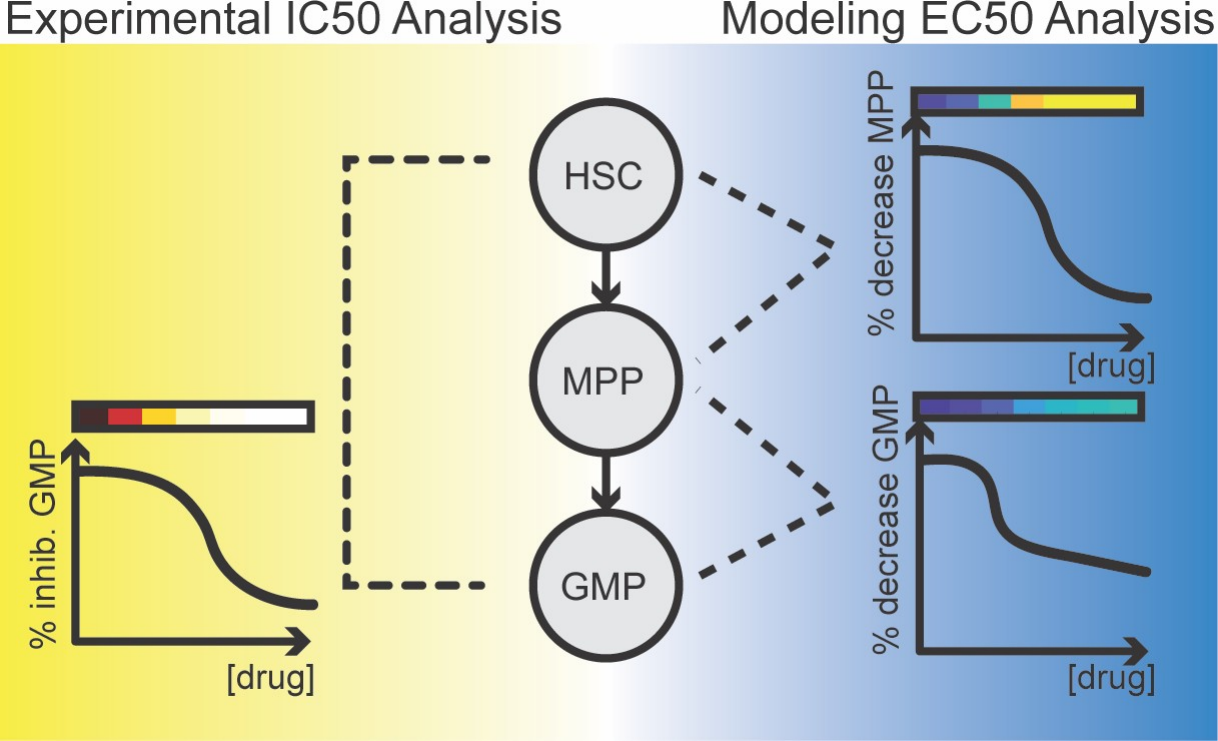
Illustration of the difference between the IC50 value assessed directly from experimental data and model-based deconvolution of mechanisms explaining downstream inhibition. The cartoon shows the percent inhibition of granulocyte-macrophage progenitors (GMP) cells at increasing doses of drug. The IC50 value derived from this experimental data represents the cumulative loss along the pathway leading to the formation of GMP, including drug impacts on the early progenitors, hematopoietic stem cells (HSC) and multi-potent progenitors (MPP). On the right, model-derived Emax and EC50 values represent the drug’s direct effect on cell-killing or anti-proliferation on individual cell types, including HSC, MPP and GMP. In the proposed modeling framework, it is possible to separate effects on a specific cell type, from propagated effects, such as loss of upstream progenitors. The color gradients above all curves represent the percent inhibition (red/yellow color) or percent decrease (blue/yellow color) and these colors are used in later figures.

We developed a quantitative systems pharmacology (QSP) model of *in vitro* hematopoiesis (hereafter referred to as *in vitro* QSP model) for quantifying the effects of multi-class anti-cancer agents on multiple cell lineages. In contrast to prior modeling work based on *in vivo* studies[12], our model is built upon a set of *in vitro* data generated using a novel multi-lineage toxicity assay (MLTA) and hence has the benefit of reduced animal use and increased throughput. In particular, we first calibrated the system parameters in the QSP model to cell kinetic proliferation data generated in the absence of any drug treatment. We subsequently generated dose-response data for drugs of interest using MLTA and fitted treatment parameters that reflect the extent and dose-dependence of drug effects per lineage. Our motivation was to understand the mechanisms of drug effects, specifically anti-proliferative and cell-killing effects, and the magnitude of these effects on hematopoietic cell lineages, from progenitors to mature cell types. Towards this goal, experimental and computational methods can complement each other, as illustrated in **Fig 1**. While an IC50 value of a drug on a particular cell type can be directly read out from the MLTA treatment data, it represents the cumulative effects on not only the cell type of interest but also all the progenitors that precede it. Through modeling and computational optimization, we can discern the contributing effects on each individual lineage to recapitulate the net observed cell count decrease. Thus, through the deconvolution of the experimental data, the *in vitro* QSP model provides an understanding into mechanistic and lineage-specific drug effects. We tested the model using drugs with known cytopenia mechanisms and used these parameters as references for considering potential cytopenic effects of novel compounds. The method has broad utility for anticipating cytopenic effects and demonstrates the value in using QSP modeling to anticipate potential safety risks in a predictive, and mechanism-driven fashion.

## Results

### An in vitro QSP model for describing hematopoiesis and cell effects in response to drug treatment

**Fig 2:**
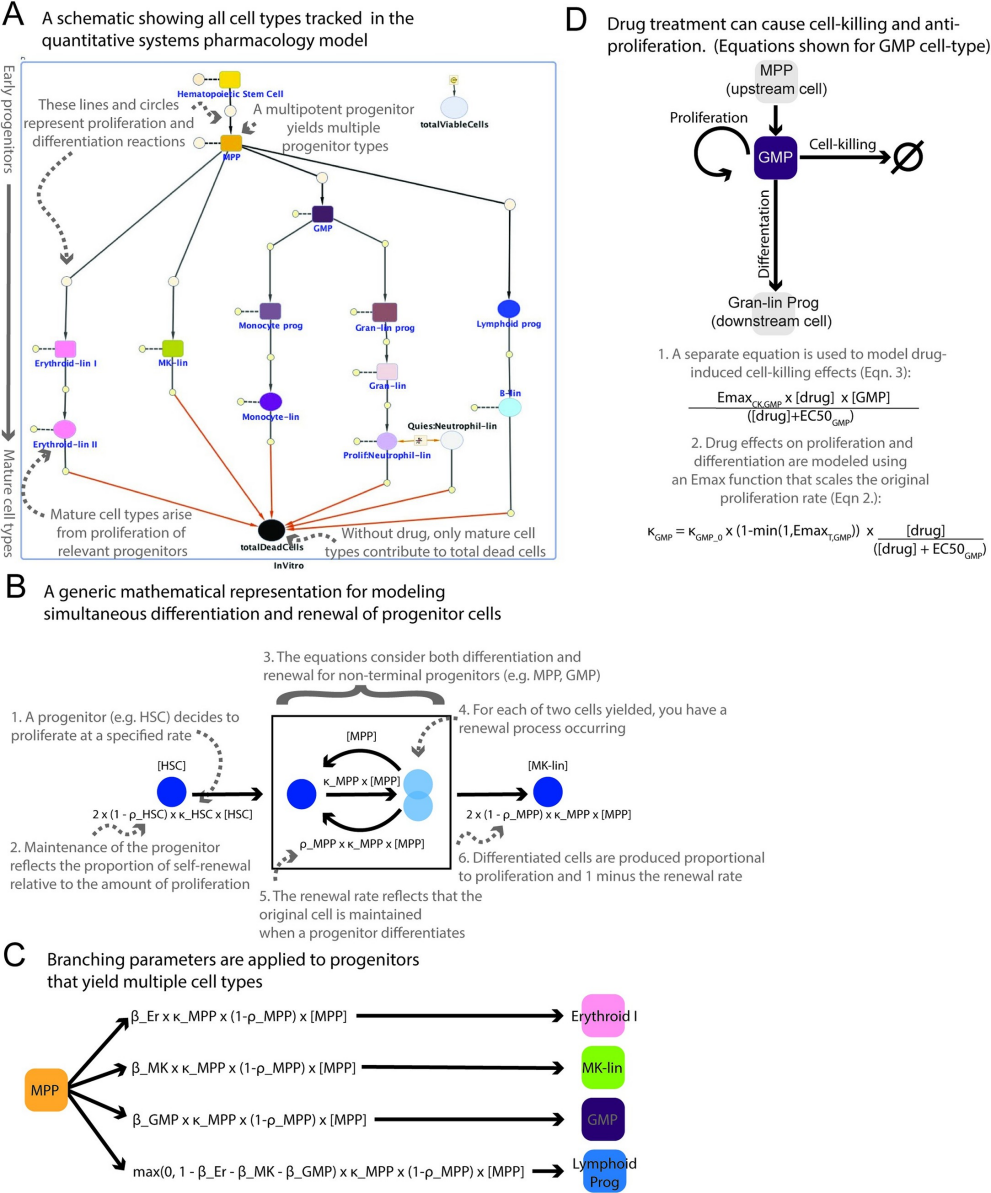
A QSP model for describing in vitro hematopoiesis, cell kinetics, and drug effects. The model contains 15 species representing 13 different cell types: hematopoietic stem cells (HSC), multi-potent progenitors (MPP), granulocyte-macrophage progenitors (GMP), monocyte progenitors (monocyte prog), monocytes (monocyte-lin), granulocyte progenitors (gran-lin prog), granulocytes (gran-lin), neutrophils, lymphoid progenitors, B-cells (B-lin), megakaryocyte cells (MK-lin), early erythroid cells (erythroid I), and late erythroid cells (erythroid II). The model also tracks total viable cells (this is the sum of the 13 hematopoietic lineages and only includes living cells), and total dead cells (we cannot distinguish between cell types in total dead cells). In addition, drug is also represented as a species in the model. The model includes a quiescent neutrophil population to adjust for the large number of neutrophils observed. This is similar to other published reports of semi-mechanistic modeling [14]. The solid arrowed lines in the diagram denote reactions resulting in the formation of products pointed to at the end of the arrow, whereas dashed lines denote reactions whereby the species in connection is both the substrate and product. Under no drug treatment, it is assumed that only the most mature cell types die, as indicated by the red arrows. For the simplicity of illustration, the anti-proliferative and killing effects of drugs on each cell type are not explicitly shown on the diagram, except for the drug-induced killing contributing towards the total number of dead cells. Shapes and colors do not encode any model information but are just meant to distinguish between cell types (**A**). The rate of increase in the number of a cell type (e.g. MPP) depends on the input flux from its predecessor cell type (e.g. HSC), proliferation flux (κ_MPP × [MPP]), as well as the fraction renewed (ρ_MPP) versus differentiated (*1 -* ρ_MPP). Note that drug effects are not represented in this diagram (**B**). Branching parameters are applied for progenitors that yield multiple cell types. All branching parameters sum to one for a given progenitor. Example shown for MPP (**C**). The model captures drug effects at the cell death reaction (“Drug cell-killing effects”) and at the proliferation reaction (“Drug anti-proliferation effects”). In this abbreviated schematic we show the drug effects on the GMP cell type and show the example equations for this species, but they are adapted for all other cell types in the model. Anti-proliferation drug effects alter the basal proliferation rate for the cell type, and this affects both proliferation and differentiation (**D**).

As we endeavored to understand the mechanistic effects of drugs on hematopoietic cell populations, we constructed an *in vitro* QSP model describing hematopoiesis, both in the control condition as well as in the presence of drug treatment (**Fig 2**) using a system of ordinary differential equations (ODEs) in MATLAB (described in Methods, full equations provided in S1 Text). The model represents the cell populations measured in the MLTA, their lineage relationships, and the processes of proliferation, differentiation vs renewal, branching, and death (see **Fig 2** for the general formulation of the equation system). In **Fig 2A**, the arrows with solid lines denote reactions, whereby the “substrate” leads to the “product” at the end of the arrow; the dashed lines denote the process of proliferation, whereby the same cell type appear as both the “substrate” and “product”. In particular, the model assumes that hematopoietic stem cells (HSCs) replenish themselves, whereas multi-potent progenitors (MPPs) numbers are maintained by the differentiation of HSCs, as well as the proliferation of MPPs. As **Fig 2A** indicates, the MPPs are assumed to give rise to all of the lineages: erythroid, megakaryocyte, monocyte, granulocyte, and lymphocyte branches. Only the most mature cell types of each lineage are assumed to die, shown with red arrows in **Fig 2A**. The model variable “totalViableCells” simply sums up all the live cells represented in the model. The full set of model cell types, parameters, flux equations, and ordinary differential equations (ODEs) are further described in (**S1 Text**). To account for differentiation and renewal, we used a mathematical formulation to describe this process (**Fig 2B**). We use a factor of two to account for the fact that cell division yields two identical cells (**Fig 2B**)–both of which can renew or differentiate. For progenitors that yield multiple cell types, branching parameters are applied to account for the probability of differentiating into a particular cell type (**Fig 2C**). These probabilities sum to one for a given progenitor. Drug effects are modeled at the cell death and proliferation reactions for all cell species (**Fig 2D**). Cell death effects are modeled using an Emax relationship. Anti-proliferation effects are modeled as affecting the basal proliferation rate of the affected cell type.

### The model explains cell kinetic data in the absence of drug treatment

**Fig 3:**
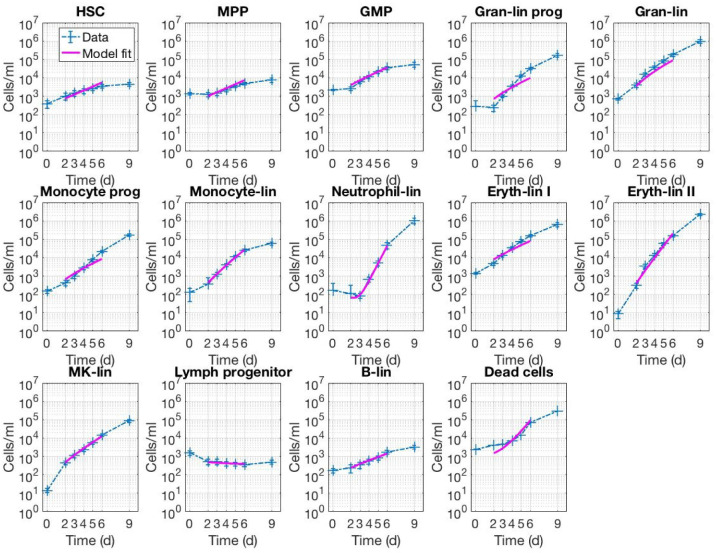
*In vitro* QSP model captures proliferation kinetics across cell types in the absence of drug treatment. Cells/mL are plotted against time (days) for 13 live cell types and the total dead cells. Error bars represent standard error of the mean of six unique donor samples. In all plots, experimental data are shown in blue dashed lines with “+” markers, and model data are shown in solid pink lines.

In order to infer the drug effects on various lineages in the MLTA (publication forthcoming) the proliferation kinetics in the control setting first needed to be understood. For this purpose, an experiment was performed to generate time-resolved measurements of cell numbers, at days 0, 2, 3, 4, 5, 6 and 9 (**Fig 3**). The kinetic data was then used to inform parameters in the *in vitro* QSP model. For the purpose of informing model parameters, the data points measured at days 0 and 9 are dropped for model fitting: the reason for dropping data at day 0 is the apparent difference in kinetics between days 0 and 2 (including a decline in counts for certain cell types, potentially due to the cells coming out of liquid nitrogen and hence not recovering until day 2) as compared to the subsequent time points; day 9 data was only generated for exploratory purposes as replating was done after the day 6 measurement, and hence also dropped for the purpose of model fitting. Using the days 2 to 6 data, a hybrid optimization approach (genetic algorithm and local search) was performed to infer a parameter set best compatible with the data (please refer to the Methods section for details). We further used profile likelihood to address parameter identifiability (Methods and S2 Text). Using the set of parameters identified, the model was able to recapitulate the kinetic data well (**Fig 3**).

### Multi-lineage concentration-response data captures cumulative lineage-specific cell responses to drug treatment

**Fig 4:**
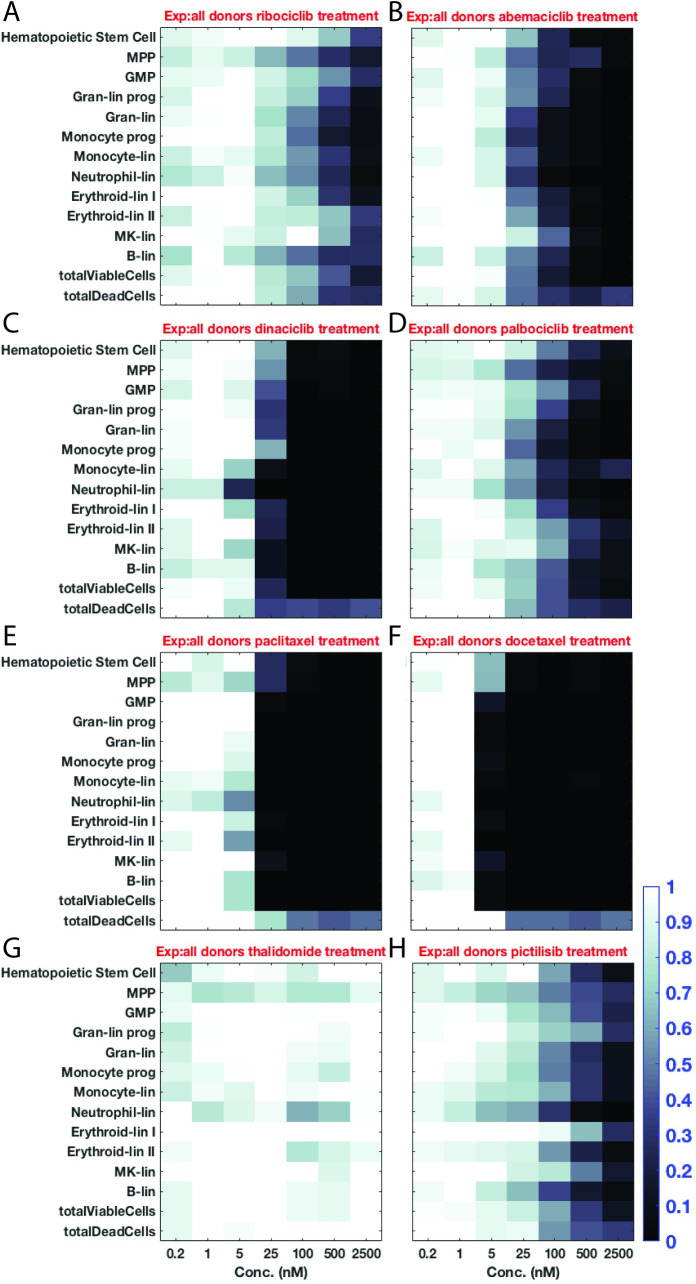
Concentration-response data captures drug effects across multiple lineages. We tested the concentration-response for ribociclib (**A**), abemaciclib (**B**), dinaciclib (**C**), palbociclib (**D**), paclitaxel (**E**), docetaxel (**F**), thalidomide (**G**), and pictilisib (**H**). Extent of shading represents relative cell counts where black or white correspond to fewer or equivalent cell counts relative to vehicle control wells respectively. In all cases, the x-axis represents doses in nM concentration (0.2, 1, 5, 25, 100, 500, 2500), and each row represents a different cell species (top: Hematopoietic Stem Cells, MPP, GMP, Gran-lin prog, Gran-lin, Monocyte prog, Monocyte-lin, Neutrophil-lin, Erythroid-lin I, Erythroid-lin II, MK-lin, B-lin, totalViableCells, and bottom: totalDeadCells).

We used the MLTA to capture dose-dependent drug effects (publication forthcoming). The 96 well assay used simultaneous differentiation of human donor CD34+ stem cells into multiple lineages and measured these lineages using flow cytometry. We tested 51 compounds (**S1 Table**). This compound set spanned multiple drug classes and contained drugs with known clinical cytopenic effects as well as novel compounds without clinical data (**Fig 4**).

The assay captured cumulative changes in numbers across cell types from a reference set of compounds with known hematopoietic toxicities. To provide context for the value of this modeling approach, we have used this reference set throughout the manuscript to describe the analysis and demonstrated usage on this reference of compounds with known hematopoietic effects before applying our method to compounds with unknown effects. Classical chemotherapies exhibited decreased numbers across cell types and this effect was relatively stronger than other drugs in this reference set (**Fig 4E & 4F**), and the cyclin-dependent kinase inhibitors (CDKis), ribociclib, abemaciclib, dinaciclib, and palbociclib (**Fig 4A, 4B, 4C & 4D**) exhibited relatively more stable cell numbers across lineages. For instance, classic chemotherapies decreased MPP cell numbers to a greater extent and at lower doses than ribociclib or palbociclib. Our negative control, thalidomide (**Fig 4G**), had the least effect on hematopoietic cell types across doses, and the phosphoinositide-3 kinase inhibitor (PI3Ki) pictilisib had a moderate reduction of cells types.

**Fig 5:**
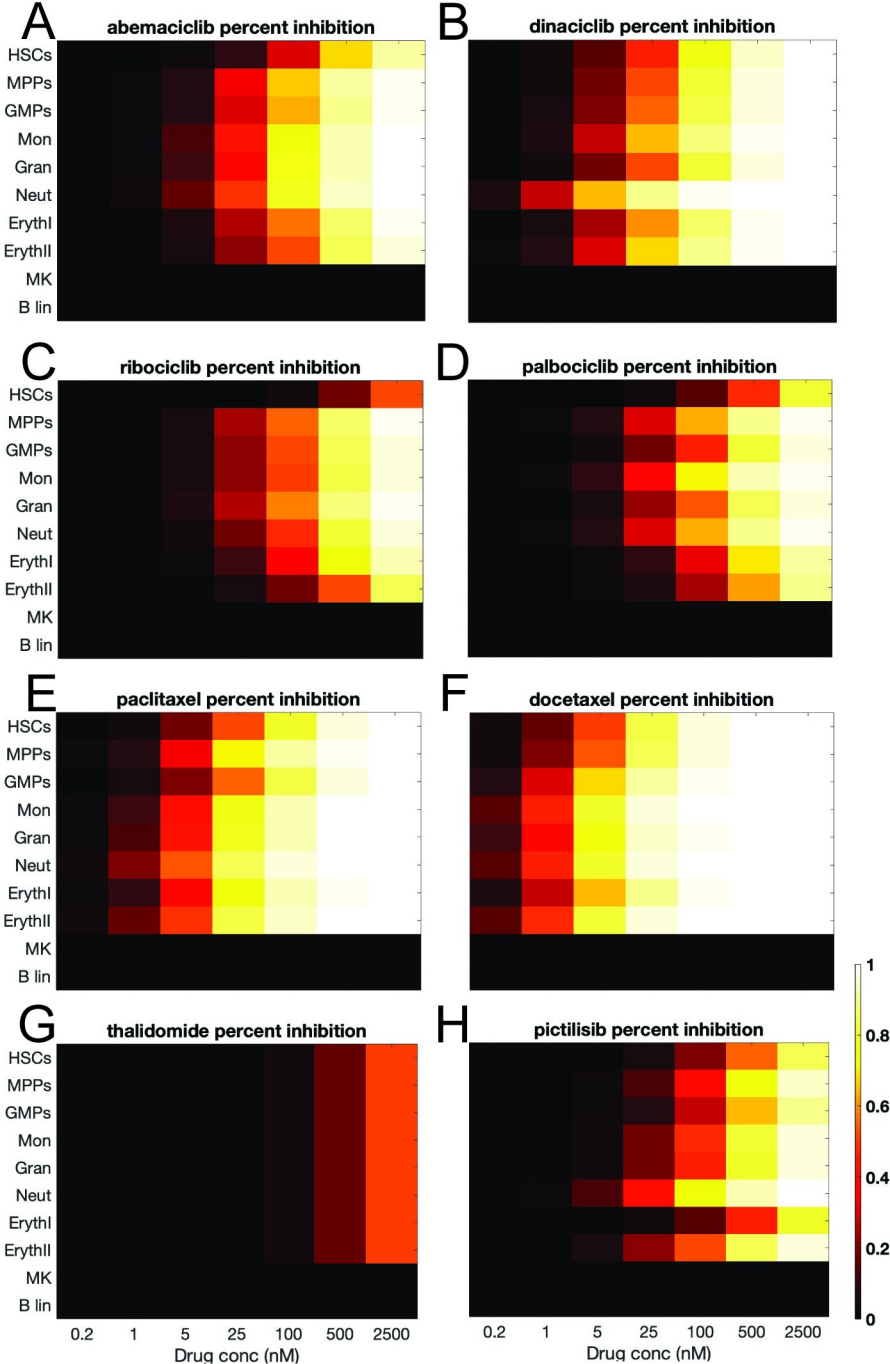
Percent inhibition plotted using IC50 values. Percent inhibition is plotted for abemaciclib (**A**), dinaciclib (**B**), ribociclib (**C**), palbociclib (**D**), paclitaxel (**E**), docetaxel (**F**), thalidomide (**G**), and pictilisib (**H**). Percent Inhibition was calculated using IC50 values fitted to the concentration-response data. Black to yellow shading represents increasing inhibition of each cell type: note that IC50s for MK- and B-lin could not be determined from the treatment data, hence these cell types are shown as having 0% suppression. The results indicate that generally a broad set of cell types are inhibited under drug treatment. In all cases, the x-axis represents doses in nM concentration (0.2, 1, 5, 25, 100, 500, 2500), and each row represents a different cell species (top: HSCs, MPPs, GMPs, Mon, Gran, Neut, ErythI, ErythII, MK, and bottom: B lin).

**Fig 6:**
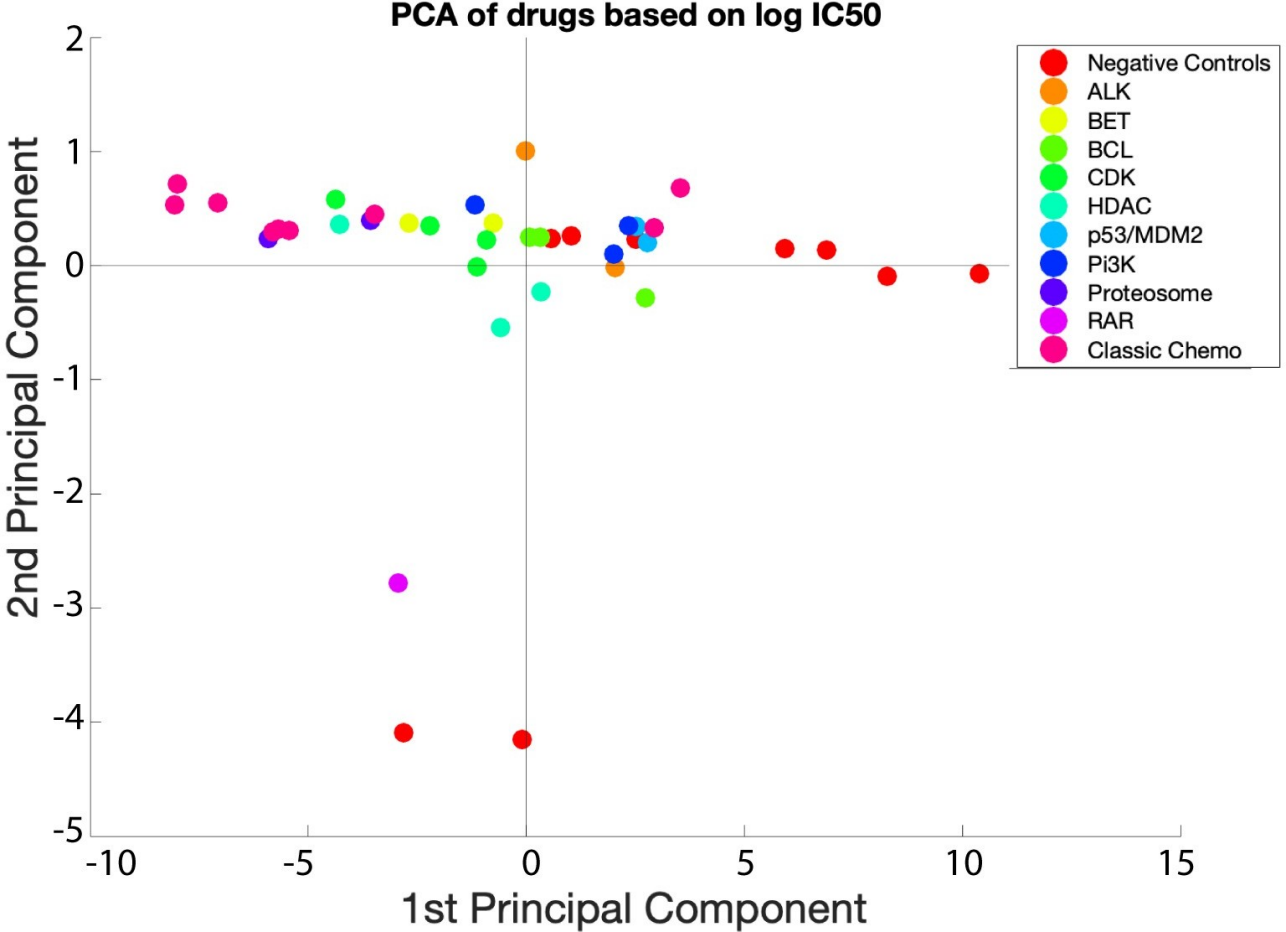
PCA of compounds using the log of experimentally derived IC50 values. A set of 46 marketed compounds, including 15 negative controls were grouped using principal component analysis. Marker color indicates drug class. The result shows that while almost all the compounds ranged between classic chemotherapies and negative controls on the first principal component, the mechanisms differentiating the compound classes cannot be well identified using the IC50 values alone.

We first applied a traditional, curve-fitting analysis to the concentration response data and identified an IC50 value for each cell type with each drug treatment. We used these IC50 values to plot normalized percent inhibition plots, based on Eq 1 (**Fig 5**). Using these relationships, the classic chemotherapies (**Fig 5E & 5F**) showed a decrease in most cell types and inhibition occurs at lower doses compared to the CDK inhibitors (**Fig 5A–5D**). The CDK inhibitors, abemaciclib, dinaciclib, ribociclib, and palbociclib, decreased cell numbers across most cell types, with dinaciclib showing a stronger decrease at lower concentrations. The negative control, thalidomide (**Fig 5G**), did not drastically decrease cell types. The PI3K inhibitor, pictilisib (**Fig 5H**) decreased most cell types. We did PCA analysis on the compounds based on the log of their experimentally derived IC50 values (**Fig 6**). Ultimately, the goal of the MLTA assay is to be predictive of clinical effects. We further explored PCA analysis as a means for identifying components that provide a “mechanistic” explanation of how these compounds affect cytopenias. In this work, we were driven to understand drug mechanisms in terms of how they affected different lineages and PCA components represented a means for investigating these mechanisms in a multivariate fashion. Compounds were generally well separated by class, and we observed that a single PCA component could explain the variability between compounds. The PCA analysis highlighted that logIC50 values do not identify much variance between compounds and suggested that this paradigm may only be partially predictive for clinical effects.

$$Percent\;Inhibition=\frac{\left[drug\right]}{[drug]+IC50}$$ Eq 1

### QSP model fitting identified quantitative, mechanistic hematopoietic effects of drug treatment on each cell type

Using the *in vitro* QSP model, we identified 26 parameters reflecting drug mechanisms for affecting hematopoietic toxicity. We adopted the approach of parsimony in explaining the observed treatment data: namely, for each drug, we fitted a single set of Emax and log(EC50) parameters for each of the 13 cell types measured (**Table 1** and full data in **S2 Table**). This is in contrast to the alternative of fitting a cell-killing Emax, a cell-killing EC50, an anti-proliferation Emax, and anti-proliferation EC50 for each drug, for each cell type (which would result in a total of 52 drug effect parameters). Goodness of fit plots and mean squared error across cell types for the reference drug set (highlighted in **Fig 4**) are included in **S1–S8 Figs**. The model was well-fitted to the experiment data for drugs in the reference set, with the exception of Thalidomide. Thalidomide was used as a negative control in the MLTA assay, and as expected, an Emax model was unable to describe these cell populations because they were relatively stable during drug treatment (**S7 Fig**).

**Table 1:** Examples of EC50 and Emax_T_ parameter values for compounds with known hematopoietic toxicity effects. Thalidomide is included as a negative control and EC50 values above the max tested dose of 2500 nM are extrapolated from model fitting.

Parameter Name	abemaciclib	dinaciclib	docetaxel	paclitaxel	palbociclib	pictilisib	ribociclib	thalidomide
EC50_HSC_	111.199	70.670	33.732	62.300	165.661	289.334	372.722	189.555
EC50_MPP_	46.123	16.681	4.706	42.378	354.142	368.700	700.610	718.402
EC50_GMP_	37.326	49.137	102.942	28.210	54.617	1.406	140.666	176.993
EC50_GranP_	33.181	548.688	127.836	98.209	0.105	93.047	21.418	128.605
EC50_Gran_	38.891	167.897	7.907	15.772	20.732	0.167	22.176	36.708
EC50_MonoP_	42.516	31.343	7.885	24.767	33.480	116.627	75.973	305.042
EC50_Mono_	46.187	28.381	9.030	30.004	39.336	1023.971	180.253	694.357
EC50_Neut_	66.213	14.199	13.699	25.092	74.956	131.773	177.785	0.302
EC50_ErythI_	29.184	25.770	8.136	15.356	42.506	190.664	88.607	424.670
EC50_ErythII_	45.082	32.781	6.299	20.180	99.537	190.999	222.347	1379.963
EC50_MK_	486.023	23.213	7.789	51.732	758.847	1070.272	2044.762	321.784
EC50_LymP_	36.507	25.655	17.633	11.696	23.977	3.805	12.339	20.077
EC50_B_	69.229	41.555	10.386	36.695	90.551	91.304	35.828	130.058
Emax_T,HSC_	0.910	1.031	1.300	1.188	0.776	0.486	0.489	0.000
Emax_T,MPP_	0.002	0.694	2.014	0.002	0.000	0.000	0.000	0.000
Emax_T,GMP_	1.034	0.001	0.000	0.001	0.884	0.071	0.760	0.000
Emax_T,GranP_	0.002	0.000	0.000	0.000	0.047	0.605	0.064	0.000
Emax_T,Gran_	0.002	2.010	2.762	2.360	0.000	0.232	0.000	0.000
Emax_T,MonoP_	0.002	0.000	1.459	1.556	0.000	1.399	0.000	0.000
Emax_T,Mono_	0.231	0.482	0.198	0.258	0.142	0.039	0.195	0.000
Emax_T,Neut_	0.235	0.281	0.232	0.214	0.184	0.165	0.139	0.011
Emax_T,ErythI_	1.024	1.115	1.726	1.466	0.000	0.000	0.001	0.000
Emax_T,ErythII_	0.033	0.071	0.016	0.071	0.118	0.136	0.108	0.000
Emax_T,MK_	0.068	0.331	0.126	0.259	0.074	0.123	0.048	0.000
Emax_T,LymP_	0.002	1.149	0.002	0.002	0.000	0.000	0.000	0.000
Emax_T,B_	0.649	0.774	1.019	0.914	0.499	0.514	0.305	0.002

In this model, we allowed drugs to have both anti-proliferation as well as cell-killing effects. However, in order to describe both effects with the most parsimonious parametric representation, we have developed the following novel formulation. The underlying assumption is that under treatment with increasing concentrations, drugs will first manifest anti-proliferation effects, followed by cell-killing effects at drug concentrations above certain thresholds that result in complete block of proliferation. The formulation relies upon a single set of Emax and log(EC50). Using the model, we estimate a total Emax value (Emax_T_) for each drug on each cell type. From Emax_T_, we can infer an antiproliferation component of Emax_T_ < = 1 (Emax_AP_ = min(1, Emax_T_)) and a cell-killing component of Emax_T_ larger than 1 (Emax_CK_ = max(0, Emax_T_ -1)).

As expected, the classic chemotherapies, docetaxel and paclitaxel, had relatively strong Emax_T_ effects. Specifically, docetaxel induced cell-killing on HSCs (Emax_T,HSC_ = 1.3, Emax_CK,HSC_ = 0.3), MPPs (Emax_T,MPP_ = 2.014, Emax_CK,MPP_ = 1.014), granulocytes (Emax_T,Gran_ = 2.762, Emax_CK,Gran_ = 1.762), monocyte progenitors (Emax_T,MonoP_ = 1.459, Emax_CK,MonoP_ = 0.459), and early erythroid cells (Emax_T,ErythI_ = 1.726, Emax_CK,ErythI_ = 0.726) and is consistent with published cell-killing effects on hematopoietic progenitors [15]. Paclitaxel induced cell-killing on HSCs (Emax_T,HSC_ = 1.188, Emax_CK,HSC_ = 0.188), granulocytes (Emax_T,Gran_ = 2.360, Emax_CK,Gran_ = 1.360), monocyte progenitors (Emax_T,MonoP_ = 1.556, Emax_CK,MonoP_ = 0.556), and early erythroid cells (Emax_T,ErythI_ = 1.466, Emax_CK,ErythI_ = 0.466) and is consistent with the known cytotoxic effects of paclitaxel [16]. Comparatively, the CDKis exhibited less cell-killing effects and more anti-proliferative effects. Abemaciclib, dinaciclib, palbociclib, and ribociclib exhibited strong anti-proliferative effects on HSCs (Emax_T,HSC_ = 0.910, 1.031, 0.776, and 0.489 and Emax_AP,HSC_ = 0.910, 1.000, 0.776, and 0.489 respectively). Dinaciclib was the only CDKi with a relatively strong cell-killing effect on granulocyte-lineage committed cells (Emax_T,Gran_ = 2.010). (**Table 1** and full data in **S2 Table**). The estimated anti-proliferative and cell-killing effects of palbociclib and dinaciclib are consistent with published mechanisms[10,17].

**Fig 7:**
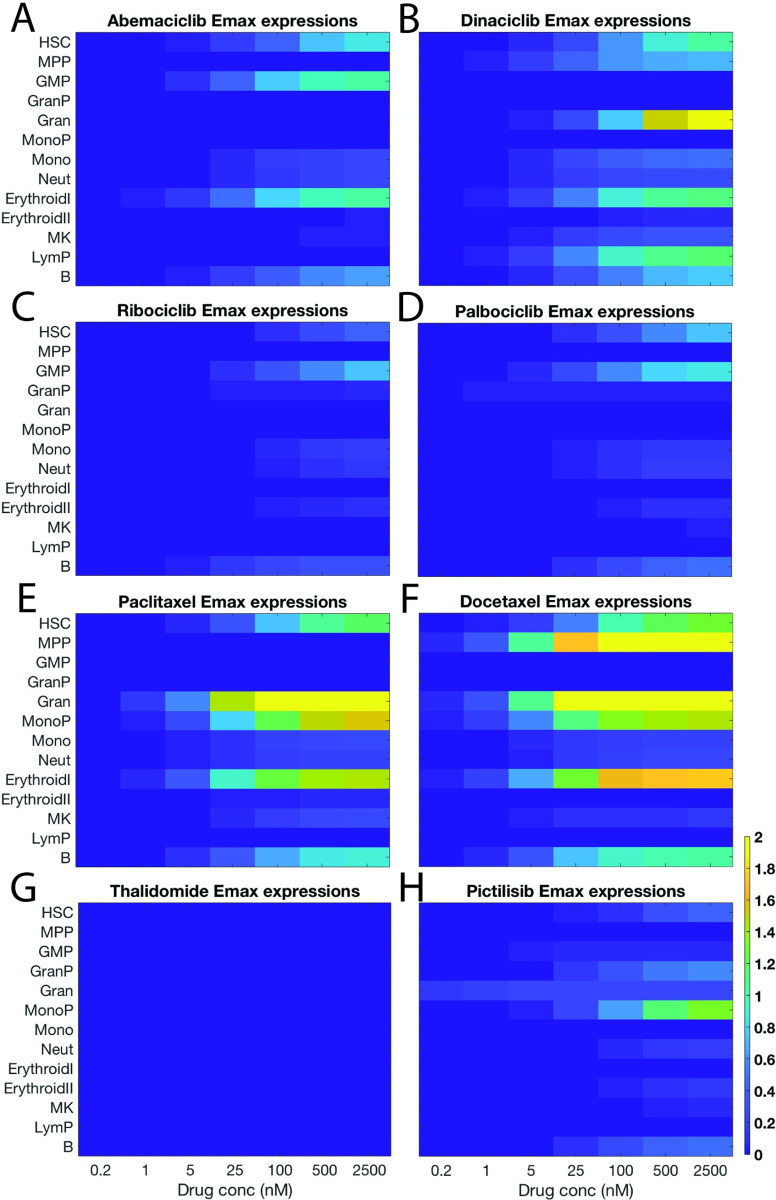
Emax_T_ expressions show relative drug effects at increasing concentrations. Emax_T_ effects are plotted against concentration for abemaciclib (**A**), dinaciclib (**B**), ribociclib (**C**), palbociclib (**D**), paclitaxel (**E**), docetaxel (**F**), thalidomide (**G**), and pictilisib (**H**). Color bars represent magnitude of Emax_T_ effect and concentrations are in nM. The results show that for select targeted therapies, the treatment data can be explained by drug effects on a parsimonious set of cell types. In all cases, the x-axis represents doses in nM concentration (0.2, 1, 5, 25, 100, 500, 2500), and each row represents a different cell species (top: HSC, MPP, GMP, GranP, MonoP, Mon, Neut, ErythroidI, ErythroidII, MK, LymP, and bottom: B lin).

We additionally plotted these results as Emax_T_ expressions (that is, ${Emax}_{T}\frac{\left[drug\right]}{[drug]+EC50}$) that illustrate drug effects at increasing concentrations (**Fig 7**). Paclitaxel, docetaxel, and dinaciclib have cell-killing mechanisms (Emax_T_ effects > 1.0), though, the classic chemotherapies, paclitaxel, and docetaxel have stronger effects at lower concentrations (**Fig 7**). For comparison, we plotted the equivalent information using the experimental IC50 values (**Fig 5**). As anticipated, the IC50s describe cumulative effects and Emax_T_ expressions identify specific cell type effects. In particular, the percent inhibition for palbociclib as shown in **Fig 5D** indicates a drop in cell counts for a block of 8 cell types ranging from HSCs, MPPs through erythrocytes; alternatively, the modeling result shown in **Fig 7D** indicates that palbociclib data can be (parsimoniously) explained by anti-proliferation effects on essentially just the HSCs and GMPs.

### Principal component analysis (PCA) separates drugs based on their Emax_T_ effects

**Fig 8:**
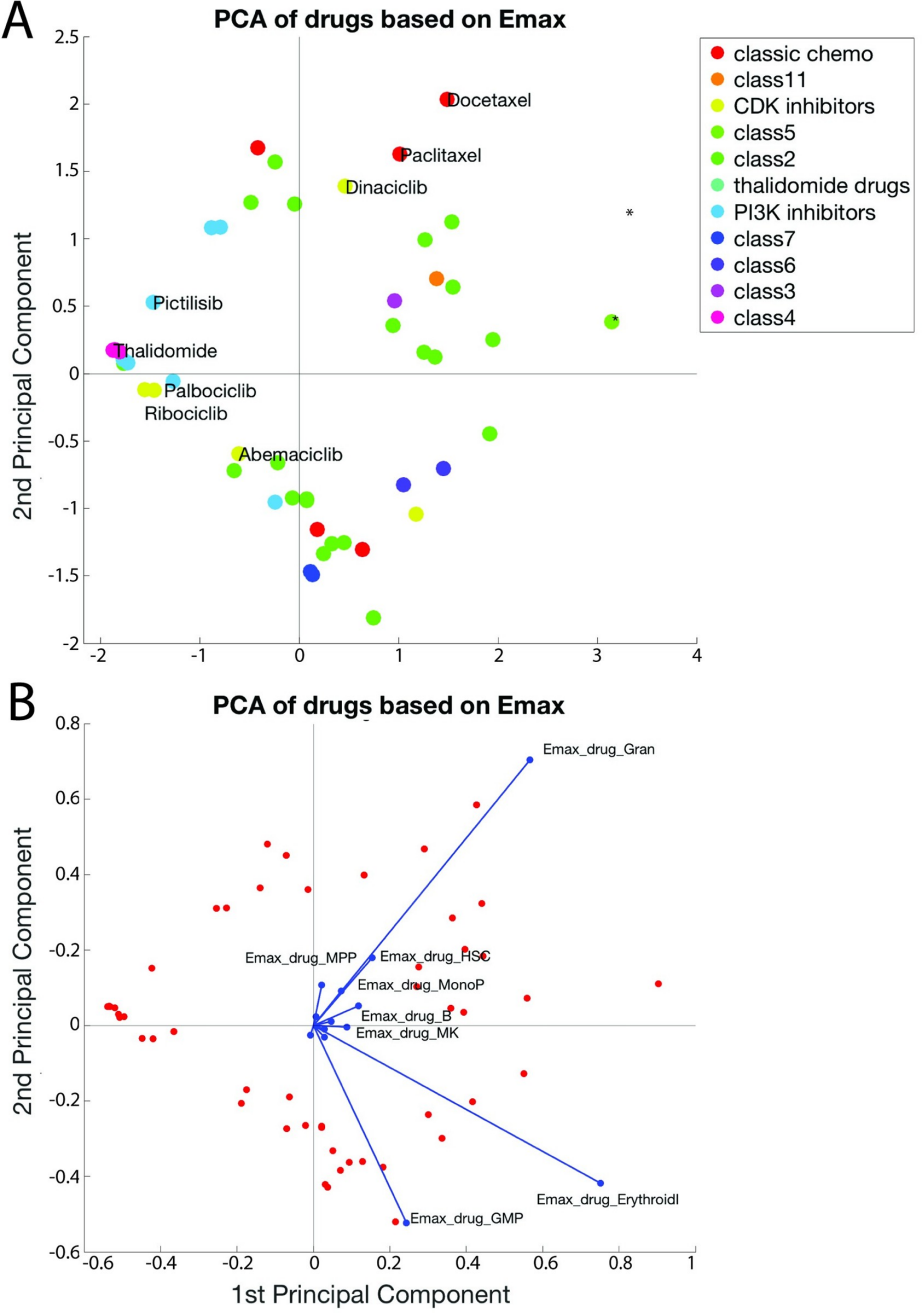
PCA separates drugs based on Emax_T_ effects. The 51 compounds are plotted in PCA space (**A**). Marker color corresponds to drug class. Drugs and variables contributing to the top two components are plotted in PCA space (**B**). Note: in figure **A** there is only one drug in class 5 and it is marked with an * to distinguish this compound from the remaining class 2 drugs.

To further understand the “mechanisms” of drug effects we again conducted PCA analysis on the Emax_T_ values generated with the full 51 drug set (**Fig 8** and **S9 Fig**) and log(EC50 values) (**S10 Fig**). The parameters most correlated (> 0.1) with PC1 and PC2 are shown in **Fig 8B**. For this analysis, we used anonymized drug class names to protect molecules in development. Indeed, both of these PCA analyses better separated the compounds compared to analysis with log(IC50) data, suggesting the ability to detect different “mechanisms” for their effects. Some drug classes were located in PCA space proximal to each other because of similar mechanisms, where there was separation between other drugs within the same class. For instance, docetaxel and paclitaxel, two microtubule inhibitors from the chemotherapy class, plotted near to each other (**Fig 8**) likely due to the large Emax parameters in regard to Granulocytes, but separate from the remaining chemotherapies, bortezomib (proteosome inhibitor), cytarabine (DNA synthesis inhibitor), and 5-FU (inhibitor of DNA synthesis). Conversely, dinaciclib was distant from other CDK inhibitors but was proximal to docetaxel and paclitaxel, as compared to all other drugs. Using the loadings plot (**Fig 8B**), it’s likely that dinaciclib was plotted distinct from other CDK inhibitors due partially to an effect on granulocytes that was not observed with other CDK inhibitors. Abemaciclib, ribociclib, and palbociclib plotted proximal to each other. Further investigation of the loadings plot (**Fig 8B**) revealed more information about the “mechanistic” effects of these drugs. Emax_T,Gran_, Emax_T,ErythroidI_, and Emax_T,GMP_ were relatively well-correlated to principal components 1 and 2 as compared to the Emax_T_ values for other cell types (**Fig 8B**) and had the greatest variance across the compounds. The coefficients for the remaining unlabeled variable names are included in **S3 Table**.

### Analysis of mechanistic effects enables consideration of experimental drug candidates

**Fig 9:**
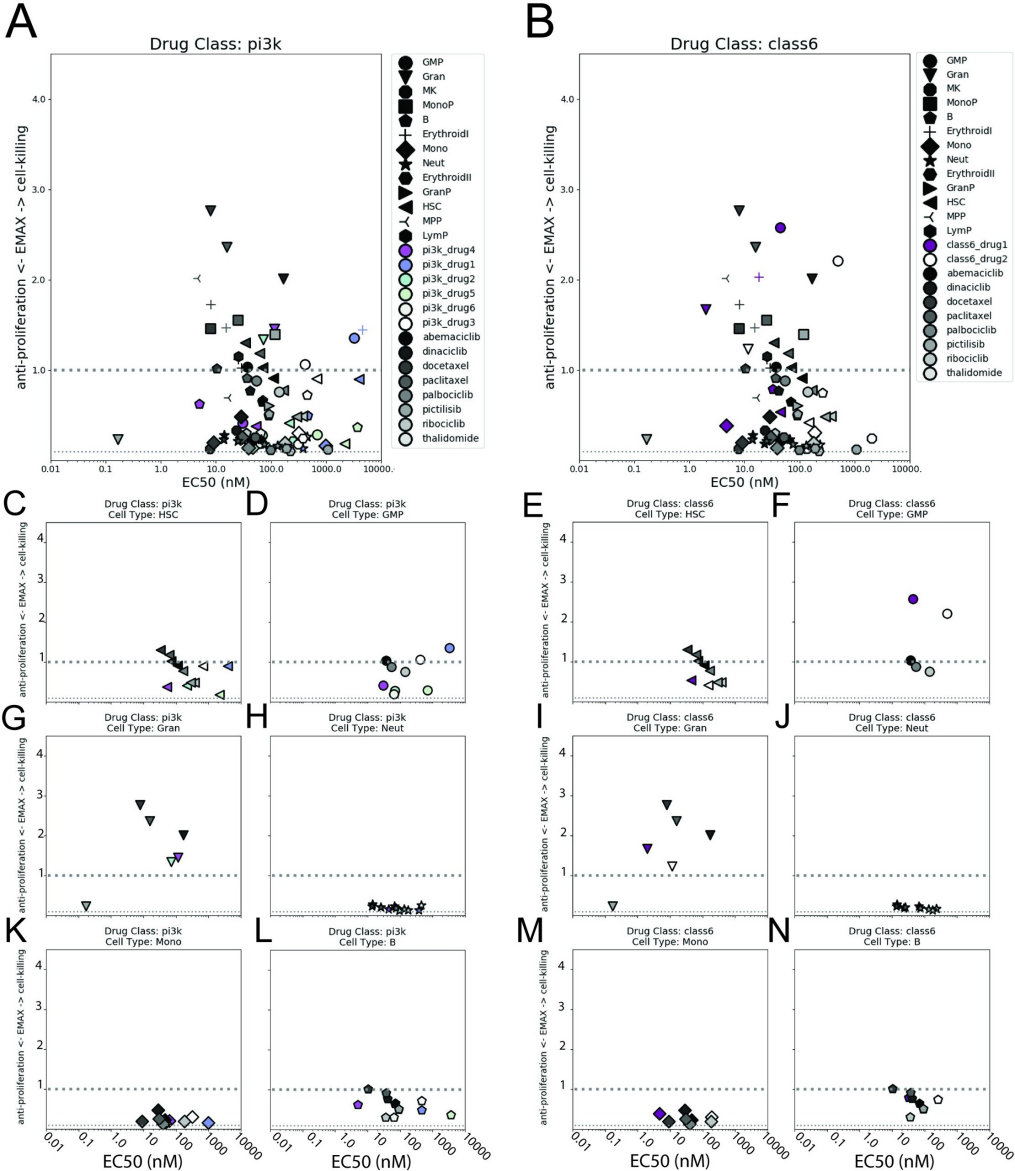
Mechanistic effects of developmental compounds per cell type compared to the reference set. Emax_T_ parameters per cell type are plotted against the EC50 values for each cell type. Marker shape represents cell type and marker shading represents either the reference set (black gradient, all figures), PI3K inhibitors (multi-color, **A**), or class 6 (purple, **B**). The same data are broken into cell type plots for PI3K inhibitors: HSCs, GMP, granulocytes, neutrophils, monocytes, B-cells (**C,D,G,H,K,L**); or for class 6 drugs: HSCs, GMP, granulocytes, neutrophils, monocytes, B-cells (**E,F,I,J,M,N**), respectively. In all plots, the x-axis represents the EC50 concentration in nM (0.01, 0.1, 1.0, 10, 100, 1000, 10000) and the y-axis represents Emax_T_ values from 0-4.The dashed line represents where Emax_T_ = 1.0. Only a subset of cell type plots is shown. Note: only compounds with Emax_T_ effects > 0.1 are plotted; compounds such as the negative control, thalidomide, had no effects above 0.1 and are not shown.

To further scrutinize novel compounds for potential hematological toxicity, we considered their magnitude of effect per cell type relative to the reference set of compounds considered in **Fig 4.** (**Fig 9**, and **S11 Fig**). Drugs in class 3 and class 6 (**S11B Fig** and **S10B Fig**) had relatively similar Emax_T_ effects compared to the reference set and these effects were shifted to higher EC50 values. The single drug in class 5 had strong cell-killing effects on early erythroid cells and GMP cells, similar to dinaciclib, docetaxel, and paclitaxel (**S11D Fig**, triangles and diamonds respectively). The class 4 compounds (**S11C Fig**) had relatively little to no Emax_T_ effects compared to the reference set. The PI3K inhibitors had similar anti-proliferative but reduced cell-killing effects as compared to the reference set (**Fig 9A**). The remaining named compounds tested (**S11E Fig**) had comparable Emax_T_ effects relative to the reference set.

## Discussion

Here we presented an approach using a QSP model of *in vitro* hematopoiesis for learning lineage-specific drug mechanisms of myelosuppression. Our ordinary differential equations model described cell kinetics in the absence of drug treatment, as well as drug effect parameter values, Emax_T_ and EC50, that explain the observed drug effects on multiple hematopoietic cell types. The major innovation in our approach is the deconvolution and mechanistic interpretation of drug effects on multiple hematopoietic lineages including early progenitors and mature blood cells. By this modeling approach, we were able to estimate the drug effect parameters (Emax_T_ and EC50) on each cell type in the hematopoiesis pathway in a manner that is independent of study treatment duration and enables model-based *in vitro* to *in vivo* translation that is underway. We expect the model-based translational approach to be more predictive of clinical outcomes, due to the fact that known differences between *in vitro* and *in vivo* hematopoiesis are captured in the mathematical models (including proliferation kinetics and feedback mechanisms mediated by cytokines such as G-CSF, EPO and TPO).

The presented approach consisted of multiple analysis stages that balance the practicality of preclinical analysis and the aim of recapitulating a complex biological system. The first computational analysis analyzed kinetic data from the MLTA assay. The assay is a robust and informative system for generating insights about cell-type specific toxicities. Our model was well-fit to the data, but we are aware of the influence of the growing conditions of the assay on the estimated parameters. For instance, the lymphocyte progenitors appear to decline in numbers. This could be due to cell death or differentiation into more mature progenitors, or perhaps limitations in generating a perfect physiological environment for this cell type. The gran-lin progenitor and total dead cell populations were only moderately fit in the kinetic model. The poor fitting for dead cells is likely due to an initial influx of dead cells after thawing that the model could not explain through kinetic parameters and the underprediction of the gran-lin progenitors is likely due to the nature of adapting *in silico* equations to *in vitro* conditions. It is possible that this population needed additional rate equations or parameters, but this complexity was outside the scope of this modeling effort. When estimating drug effects, we used human donor cells that also have some variability, and thus, a range of variability/uncertainty in the estimated parameters. We sought robust drug effect parameter estimates by using a relatively high number of donor samples to minimize these effects and to perform an analysis that is practical in a preclinical setting. We employed an Emax model that was well-suited to our experimental system, and specifically endeavored to discover Emax parameters that described the magnitude of drug effect on each cell type. We acknowledge that these conditions are not appropriate for every system and we acknowledge that there are other Emax model formulations. Specifically, we assumed that a drug must exert anti-proliferation effects before cell killing. We made this assumption to reduce the total number of parameters and because mechanistically, CDK-inhibitors are already known to have antiproliferation effects [10]. However, the model could easily be extended to have separate cell killing and anti-proliferation parameters. Additionally, for systems where the modelers anticipate very large EC50 values, linear drug effects may be appropriate, and these effects can be encoded in the model.

We used these parameters with multivariate and comparative analyses across compounds to anticipate if and the extent of hematological toxicity of novel compounds. More specifically, we selected an example set and compared parameters of novel compounds in development to the parameters associated with the sample set. We anticipated this modeling and interpretation paradigm to generally be valuable for early stage development decisions. Multivariate analysis enabled comparison of novel compounds based on their complete set of Emax_T_ values. Here we highlighted that dinaciclib is broadly more similar to the classic chemotherapies than to other CDK inhibitors. Further, cell-type-specific analysis of drug classes compared to the reference set enabled cell-type specific consideration of within-class drugs. This analysis could provide support for discriminating one drug class over another, or rank-order compounds within the same class based on their toxicity profiles. For instance, the PI3K and class 4 compounds had favorable toxicity parameters compared to the reference set where the single agent in class 5 had much stronger cell-killing effects than the reference set. The platform is flexible, and the reference drug set can be modified to reflect compounds and toxicity effects specific to a development program.

Many compounds are known to be cytotoxic to hematopoietic cells, yet, cytopenias measured in the blood do not reflect the full extent of damage to the hematopoietic system[18]. Understanding of mechanisms for myelosuppression would further inform first-in-human trials and help anticipate possible adverse-event mitigating strategies such as appropriate cytokine therapy by increasing evidence-based practices [3,11]. For instance, cytokine therapy is already employed for patients receiving anti-cancer therapy. Understanding how and where the drug affects the hematopoietic lineages will help inform whether or not a specific cytokine therapy might mitigate potential safety concerns; for instance, a drug that results in the loss of hematopoietic stem cells would have a different clinical risk implication from one that depletes the granulocyte lineage alone.

A mechanistic understanding of lineage-specific drug effects could inform further translational modeling. Specifically, we are actively developing an *in vivo* clinical model that translates *in vitro* drug parameters to simulate multiple cytopenias, including thrombocytopenia, neutropenia, and anemia. Thus, while we are eager to translate these findings to clinical applications, it was not within the scope of this work to further establish relationships between *in vitro* and *in vivo* parameters. Currently, our *in vitro* parameters can inform a related *in vivo* ODE model of hematopoiesis to predict clinical cytopenias in the presence of drug treatments. This model would additionally need pharmacokinetic parameters and models to incorporate clinically relevant drug exposures. Pharmacokinetic data is published for many anticancer therapies, making it possible to develop a clinical model and eventually create more predictive paradigms for anticipating clinical cytopenias. This *in vivo* model could inform optimal dosing schedules considering multiple cytopenia effects and ultimately inform personalized treatment approaches [18]. This model contains similarities to the Friberg model [8] such as multiple maturation compartments. Yet our priority will be to compare the model to translational data instead of benchmarking on this foundational model.

## Materials, methods, and model

### In vitro multilineage hematopoietic toxicity assay culture system

A custom optimized cytokine cocktail was developed using commercially available cytokines (Peprotech, New Jersey) to facilitate simultaneous multi-lineage differentiation and self-renewal of freeze-thawed primary human bone marrow-derived CD34+ cells in SFEM II media (cells and media from StemCell Technologies, Vancouver). Cultures were carried out in ultra-low attachment 96-well plates (Corning, New York) with test article added on day 0 and cultured at 37°C, 85% relative humidity, and 5% CO2 for six days before interrogating drug impact via 17-parameter flow cytometry.

### Flow cytometry panel and analysis

The flow cytometry panel used to define hematopoietic subsets was composed of CD34-PECy7, CD90-PECY5, CD38-AF488, CD371-PE, CD45Ra-BV421, CD14-BV605, CD42b-AF647, CD10-PEDazzle594, CD235a-PerCPCy5.5 (BioLegend, California), CD15-BUV395, CD41a-APCH7, CD71-BV786 (BD Biosciences, California), and DAPI for dead cell exclusion (ThermoFisher, California). In short, Day 6 cells were harvested, FcR-blocked (FcR binding inhibitor, ThermoFisher), surface-stained, then analyzed in a buffer containing TruCount Control Beads using a BD LSR Fortessa SORP fitted with a high-throughput sampler (BD Biosciences, California). Data was analyzed in Diva 6.0.2 and exported to CSV where after all cell data was transformed to cell per mL then normalized on a per-donor basis to untreated control wells to determine drug impact.

### Measuring drug-free cell kinetics

Six replicate samples of human donor CD34+ stem cells were seeded in 96 well plates and exposed to a customized cytokine cocktail to promote differentiation (publication forthcoming). Cells were maintained in culture for six days and cell lineages were measured using flow cytometry as described above. We further used profile likelihood to address the identifiability of these parameters. This analysis and results are explained in **S2 Text**.

### Model simulation and calibration to cell kinetic data without treatment

We implemented the model using the Simbiology toolbox in MATLAB, version 2019A. The flux equations, ODEs, model parameters and model cell types are further described in **S1 Text**. The model tracks 13 live cell types: hematopoietic stem cells (HSCs), multi-potent progenitors (MPPs), granulocyte-macrophage progenitor (GMPs), granulocyte progenitor (Gran-lin prog), granulocytes (Gran-lin), monocyte progenitors (monocyte prog), monocytes (monocyte-lin), neutrophils (neutrophil-lin), early erythroid cells(eryth I), late erythroid cells (eryth II), MK cells (MK-lin), lymphocyte progenitors (LymP) and B cells (B-lin); the total number of dead cells is also described (totalDeadCells). The ODE system describes the various steps of differentiation, proliferation, and renewal of hematopoietic stem cells into mature lineages. The most mature cell type of each lineage (that is: eryth II, MK-lin, monocyte-lin, neutrophil-lin and B-lin) are assumed to die, at a uniform rate (kDeath). In summary, the system parameters of the model included cell-type specific renewal rates (denoted with ρ for each cell type), proliferation rates (denoted with κ for each cell type), branching rates (denoted with β for each cell type), and a uniform death rate (denoted with δ). All δ parameters are assumed to be zero for non-terminal cell types in the absence of drug treatment. For the LymP cell type, we did not include a renewal parameter because this parameter wasn’t necessary for describing the kinetic data; it appears that these cells are differentiating but not proliferating in the MLTA assay. We have used a generic representation for equations in the figures and methods section and have provided a mapping to the specific parameter names as implemented in MATLAB (**S3 Text**). We have additionally included the MATLAB SimBiology file which is accessible through GitHub (https://github.com/jenwilson521/Multilineage_InvitroHem_Model).

The 27 system parameters of the model as well as the initial cell counts (at time = 0) for each of the 13 cell types and total dead cells modeled were fitted to describe the mean of the kinetic data over the 6 donors. In particular, a hybrid optimization approach combining genetic algorithm with local search was used (as implemented in the MATLAB function, ga) to obtain an optimal parameter set that best explain the experimental data. For the algorithm settings, the maximum number of generations (MaxGenerations) and the population size (PopulationSize) were set to 50 and 500 respectively. Appropriate lower and upper bounds ([LB, UB]) were set on the parameter values and initial conditions, as follows:

- Renewal rates: [0.5, 1] for HSCs and [0, 0.5] for all other proliferating cell types
- Branching rates: [0.001, 1] and summing up to 1 at each branching step
- Proliferation rates: [0, 4/log(2)]
- Death rate: [0, 2]
- Initial conditions: [0.1, 1] x measured cell counts @ t = 0

### Multi-lineage toxicity assay (MLTA) using anti-cancer therapies

Donor material was collected and cultured as described above. The number of replicate donor samples per drug ranged from 2–7 and varied per drug (**S1 Table)**. Drug treatment was added at time 0 and cells were maintained in culture for 6 days. Concentrations tested for each drug compound are contained in **S1 Table**. Concentrations were selected to span a range that is clinically useful. Following treatment, cell populations were measured using flow cytometry. We extracted cell counts from flow cytometry data, normalized to bead counts, and corrected for well volume.

### Data normalization and pooling across donors

Each well was normalized to the average of six vehicle control wells. For drugs that were tested across multiple runs of the assay, vehicle-normalized donor data were pooled, and we used the average of these pooled data for further modeling. Normalized data used as model inputs are included in **S1 Table**.

### Creating an in vitro model for hematopoiesis in the presence of drug treatment

We implemented drug effects using an Emax model for the drug’s effect on each cell type. The 13 maximum effect parameters (Emax_T,C_) are unitless and can vary between 0 and 2 and where a subscript, C, represents one of the previously described 13 cell types. We adapted a traditional Emax model using the equations below. We interpreted Emax_T_ values less than or equal to one as anti-proliferative, and Emax_T_ values greater than one as indicating a cell-killing mechanism in addition to anti-proliferation. The relationships for these effects are included in the model using the following equations. Eq 2 describes the drug anti-proliferative effect in the net proliferation rate for a given cell type. Eq 3 describes the cell-killing Emax_CK_ effect.

### Anti-proliferation:

$$\;\kappa_{C}=\kappa_{C0}\times \left(1-\min\left(1,{Emax}_{T,C}\right)\times (\frac{\left[drug\right]}{EC50_{C}+[drug]})\right)$$ Eq 2

Where:

$$\kappa_{C}=post-treatment\;proliferation\;rate\;for\;cell\;type,C$$

$$\kappa_{C0}=basal\;proliferation\;rate\;for\;cell\;type,C$$

$${Emax}_{T,C}=total\;Emax\;parameter\;relative\;to\;cell\;type,C$$

$${logEC50}_{C}=\log\;of\;EC50\;relative\;to\;cell\;type,C$$

$${EC50}_{C}=\exp\left(log{EC50}_{C}\right)$$

### Cell-killing:

$$\left(\frac{Emax_{CK,C}\times [drug]}{[drug]+{EC50}_{C}}\right)\times [C]$$ Eq 3

Where:

$$Emax_{CK,C}=\max\left(0,{Emax}_{T,C}-1\right)$$

[C]=concentrationofcelltype,C

In the above equations, *Emax*_*CK*,*C*_ has units of (1/day) and [C] represents the concentration of the relevant cell type.

### Extracting EC50 and EMAX effects to explain drug myelosuppression mechanisms

For estimating mechanistic parameters, we fitted the normalized concentration-response data for each drug across 13 cell types: hematopoietic stem cells (HSCs), multi-potent progenitors (MPPs), granulocyte-macrophage progenitor (GMPs), granulocyte progenitor (Gran-lin prog), granulocytes (Gran-lin), monocyte progenitors (monocyte prog), monocytes (monocyte-lin), neutrophils (neutrophil-lin), early erythroid cells (Eryth I), late erythroid cells (Eryth II), MK cells (MK-lin), and B cells (B-lin), via the optimization procedure (further described below), for each drug in question we identified a set of 26 parameters: 13 total Emax_T_ parameters and 13 total EC50 parameters (one Emax_T_ and one EC50 parameter per cell type).

Towards the estimation of mechanistic parameters, we considered different formulations: (a) weighting the objective value to prioritize fitting dead cells and (b) using regularization methodology to encourage a parsimonious solution. We assessed the contributions of these formulations using mean-squared error and goodness of fit plots for drugs with known mechanisms. We discovered that weighting the distance between the observed and estimated value for the dead cell populations by a factor of two was necessary to fit known cell-killing and anti-proliferation mechanisms. Further, we used L1 regularization to penalize parameters that were close to zero to help identify a parsimonious set of parameters to explain drug effects. That is, we minimized the weighted objective function as shown in Eq 4, where $M_{C}^{data}$ and $M_{C}^{model}$ are the concentration-response data and model simulation for cell type *C* respectively, with the regularization parameter set to λ = 0.1 and weights set to *w*_totalDeadCells_ = 2, and *w*_*C*_ = 1 otherwise. Thus, the model was incentivized to reduce any near-zero parameters to zero and explain drug effects through a parsimonious set of Emax_T_ and EC50 parameters. This regularization approach selects for the simplest solution given the data.

$$objective\;function=\sum_{C}∥w_{C}\times (M_{C}^{model}-M_{C}^{data}){∥}^{2}+\lambda \times \sum_{C}\left|{Emax}_{T,C}\right|$$ Eq 4

We solved the minimization problem of Eq 4 using a hybrid optimization approach combining genetic algorithm with local search (as implemented in the MATLAB function, ga). For the algorithm settings, the maximum number of generations (MaxGenerations) and the population size (PopulationSize) were set to 30 and 300 respectively. We fitted the logEC50 parameters (rather than EC50 values) to improve the ability of optimization algorithms in finding the best fitting parameter values. The following lower and upper bounds ([LB, UB]) were set on the drug effect parameters:

- Emax_*T*,*C*_: [0, 2]
- *logEC*50_*C*_: [-2.3, 8.5]

### Principal component analysis (PCA) of 51 compound set

We conducted PCA of the drug parameters estimated from the 51 compounds using MATLAB version 2019A (selecting the singular value decomposition algorithm and other default settings), with the aim of reducing the dimensionality of the inferred parameters and helping to identify patterns within compound classes. Because all of our Emax_T_ values and logEC50 values were on the same scale, our analysis required no further data scaling. We tested both PCA using only Emax_T_ effects and only logEC50 parameters.

### Mechanistic plots of lineage specific drug effects

We created cell-type specific plots using Python version 2.7.16.

## Supporting information

S1 Table *S1Table_drugs_and_doses*.*xlsx*: This contains drug names or anonymized names and the doses tested in the MLTA. This is the raw data for the paper.
(XLSX)

S2 Table *S2Table_drug_emax_logEC50_params*.*xlsx*: For all drugs, this contains parameters fitted from the QSP model. (XLSX)

S3 Table *S3Table_coefficients_from_PCA*.*xlsx*: This contains coefficients from the PCA analysis for all drugs. (XLSX)

S1 Text *S1Text_in_vitro_equations*.*pdf*: This contains all equations, rules, and parameters as used in MATLAB Simbiology to implement the ODE model. (PDF)

S2 Text *S2Text_ProfileLikelihood_HemeToxQSP*.*pdf*: This is a Supplementary analysis that addresses parameter identifiability. (PDF)

S3 Text *S3Text_parameter_table*.*pdf*: This is a table that maps between parameter names used in explanatory figures and the methods section and the parameter names implemented in ODEs for MATLAB. (PDF)

S1 Fig Goodness of fit across cell types for abemaciclib. For each cell type and total live and viable cells, normalized cell counts from experimental data (open circles) and simulated results (solid line) are plotted against concentration (nM). Additionally, each plot includes the mean squared error of the difference between experimental and data plotted with the estimated Emax_T_ effects.
(PNG)

S2 Fig Goodness of fit across cell types for dinaciclib. For each cell type and total live and viable cells, normalized cell counts from experimental data (open circles) and simulated results (solid line) are plotted against concentration (nM). Additionally, each plot includes the mean squared error of the difference between experimental and data plotted with the estimated Emax_T_ effects.
(PNG)

S3 Fig Goodness of fit across cell types for palbociclib. For each cell type and total live and viable cells, normalized cell counts from experimental data (open circles) and simulated results (solid line) are plotted against concentration (nM). Additionally, each plot includes the mean squared error of the difference between experimental and data plotted with the estimated Emax_T_ effects.
(PNG)

S4 Fig Goodness of fit across cell types for ribociclib. For each cell type and total live and viable cells, normalized cell counts from experimental data (open circles) and simulated results (solid line) are plotted against concentration (nM). Additionally, each plot includes the mean squared error of the difference between experimental and data plotted with the estimated Emax_T_ effects.
(PNG)

S5 Fig Goodness of fit across cell types for docetaxel. For each cell type and total live and viable cells, normalized cell counts from experimental data (open circles) and simulated results (solid line) are plotted against concentration (nM). Additionally, each plot includes the mean squared error of the difference between experimental and data plotted with the estimated Emax_T_ effects.
(PNG)

S6 Fig Goodness of fit across cell types for paclitaxel. For each cell type and total live and viable cells, normalized cell counts from experimental data (open circles) and simulated results (solid line) are plotted against concentration (nM). Additionally, each plot includes the mean squared error of the difference between experimental and data plotted with the estimated Emax_T_ effects.
(PNG)

S7 Fig Goodness of fit across cell types for thalidomide. For each cell type and total live and viable cells, normalized cell counts from experimental data (open circles) and simulated results (solid line) are plotted against concentration (nM). Additionally, each plot includes the mean squared error of the difference between experimental and data plotted with the estimated Emax_T_ effects.
(PNG)

S8 Fig Goodness of fit across cell types for pictilisib. For each cell type and total live and viable cells, normalized cell counts from experimental data (open circles) and simulated results (solid line) are plotted against concentration (nM). Additionally, each plot includes the mean squared error of the difference between experimental and data plotted with the estimated Emax_T_ effects.
(PNG)

S9 Fig Percent variance explained by each principal component. The percent variance(left axis) and cumulative variance (right axis) are plotted against the top six components (x-axis) for PCA analysis of Emax_T_ values.
(PNG)

S10 Fig Principal component analysis of drugs based on the logEC50 of their effects. The 51 compounds are plotted in PCA space (**A**). Marker color corresponds to drug class. Drugs and variables contributing to the top two components are plotted in PCA space (**B**). Note: in figure **A** there is only one drug in class 5 and it is marked with an * to distinguish this compound from the remaining class 2 drugs. Variance explained by each principal component is plotted in (**C**).
(PNG)

S11 Fig Mechanistic effects of developmental compounds per cell type compared to the sample set. Emax parameters per cell type are plotted against the EC50 values for each cell type. Marker shape represents cell type and marker shading represents either the sample set (black gradient, all figures) or drugs in class 2 (blues, **A**), class 3 (purples, **B**), class 4 (browns, **C**), class 5 (orchids, **D**), class 7 (green, **E**), or all other drugs (reds, **F**). The dashed line represents where Emax = 1.0.
(PNG)
