# Supplementary material for: An in vitro quantitative systems pharmacology approach for deconvolving mechanisms of drug-induced, multilineage cytopenias
Source: PLoS Comput Biol. 2020 Jul 23;16(7):e1007620. doi: 10.1371/journal.pcbi.1007620 (PMC7402526; doi:10.1371/journal.pcbi.1007620)
Supplement: S1 Text — (PDF) [file pcbi.1007620.s004.pdf]

SimBiology Model: Multilineage_InvitroHem

Fluxes:

1. reaction_1 = kpro_HSC*[Hematopoietic Stem Cell]
2. reaction_2 = kpro_HSC*(1-renewal_HSC)*[Hematopoietic Stem Cell]
3. reaction_3 = kpro_MPP*MPP
4. reaction_5 = kbranch_GMP*kpro_MPP*(1-renewal_MPP)*MPP
5. reaction_6 = max(0,1-kbranch_Erythroid-kbranch_MK-kbranch_GMP)*kpro_MPP*(1-renewal_MPP)*MPP
6. reaction_8 = kbranch_Mono*kpro_GMP*(1-renewal_GMP)*GMP
7. reaction_9 = kpro_MonoP*(1-renewal_MonoP)*[Monocyte prog]
8. reaction_10 = kpro_GranP*(1-renewal_GranP)*[Gran-lin prog]
9. reaction_12 = kpro_ErythroidI*(1-renewal_ErythroidI)*[Erythroid-lin I]
10. reaction_13 = kbranch_MK*kpro_MPP*(1-renewal_MPP)*MPP
11. reaction_14 = kbranch_Erythroid*kpro_MPP*(1-renewal_MPP)*MPP
12. reaction_19 = kdiff_lym*[Lymphoid prog]
13. reaction_21 = kpro_GMP*GMP
14. reaction_22 = kpro_MonoP*[Monocyte prog]
15. reaction_24 = kpro_MK*[MK-lin]
16. reaction_25 = kpro_ErythroidI*[Erythroid-lin I]
17. reaction_26 = kpro_ErythroidII*[Erythroid-lin II]
18. reaction_28 = kpro_Gran*[Gran-lin]
19. reaction_30 = kpro_Mono*[Monocyte-lin]
20. reaction_31 = kpro_Gran*(1-renewal_Gran)*[Gran-lin]
21. reaction_15 = (1-kbranch_Mono)*kpro_GMP*(1-renewal_GMP)*GMP
22. reaction_4 = kpro_GranP*[Gran-lin prog]
23. reaction_32 = kpro_B*[B-lin]
24. reaction_16 = kpro_Neut*[Prolif:Neutrophil-lin]
25. reaction_7 = (kDeath+(Emax_cellkill_ErythroidII*drug)/(drug+EC50_unit*exp(log_EC50_ErythroidII)))*[Erythroid-lin II]
26. reaction_11 = (kDeath+(Emax_cellkill_MK*drug)/(drug+EC50_unit*exp(log_EC50_MK)))*[MK-lin]
27. reaction_17 = (kDeath+(Emax_cellkill_Mono*drug)/(drug+EC50_unit*exp(log_EC50_Mono)))*[Monocyte-lin]
28. reaction_18 = (kDeath+(Emax_cellkill_Neut*drug)/(drug+EC50_unit*exp(log_EC50_Neut)))*[Prolif:Neutrophil-lin]
29. reaction_20 = (kDeath+(Emax_cellkill_B*drug)/(drug+EC50_unit*exp(log_EC50_B)))*[B-lin]
30. reaction_23 = (kDeath+(Emax_cellkill_Neut*drug)/(drug+EC50_unit*exp(log_EC50_Neut)))*[Quies:Neutrophil-lin]
31. Kill_EryI = (Emax_cellkill_ErythroidI*drug)/(drug+EC50_unit*exp(log_EC50_ErythroidI))*[Erythroid-lin I]
32. Kill_MPP = (Emax_cellkill_MPP*drug)/(drug+EC50_unit*exp(log_EC50_MPP))*MPP
33. Kill_HSC = (Emax_cellkill_HSC*drug)/(drug+EC50_unit*exp(log_EC50_HSC))*[Hematopoietic Stem Cell]
34. Kill_MonoP = (Emax_cellkill_MonoP*drug)/(drug+EC50_unit*exp(log_EC50_MonoP))*[Monocyte prog]

2. Kill_GMP = (Emax_cellkill_GMP*drug)/(drug+EC50_unit*exp(log_EC50_GMP))*GMP
3. Kill_GranP = (Emax_cellkill_GranP*drug)/(drug+EC50_unit*exp(log_EC50_GranP))*[Gran-lin prog]
4. Kill_Gran = (Emax_cellkill_Gran*drug)/(drug+EC50_unit*exp(log_EC50_Gran))*[Gran-lin]
5. Kill_LymP = (Emax_cellkill_LymP*drug)/(drug+EC50_unit*exp(log_EC50_LymP))*[Lymphoid prog]

Repeated Assignments:

1. [Neutrophil-lin] = [Prolif:Neutrophil-lin]+[Quies:Neutrophil-lin]
2. totalViableCells = [Neutrophil-lin]+[Lymphoid prog]+[Monocyte-lin]+[Erythroid-lin I]+[Erythroid-lin II]+[MK-lin]+[Gran-lin prog]+MPP+[Hematopoietic Stem Cell]+GMP+[Monocyte prog]+[B-lin]+[Gran-lin]
3. viability = totalViableCells/(totalViableCells+totalDeadCells)

ODEs:

1. d([Lymphoid prog])/dt = 2*reaction_6 - reaction_19 - Kill_LymP
2. d([Monocyte-lin])/dt = 2*reaction_9 + reaction_30 - reaction_17
3. d([Erythroid-lin II])/dt = 2*reaction_12 + reaction_26 - reaction_7
4. d([MK-lin])/dt = 2*reaction_13 + reaction_24 - reaction_11
5. d([Erythroid-lin I])/dt = -2*reaction_12 + 2*reaction_14 + reaction_25 - Kill_EryI
6. d(MPP)/dt = 2*reaction_2 + reaction_3 - 2*reaction_5 - 2*reaction_6 - 2*reaction_13 - 2*reaction_14 - Kill_MPP
7. d([Hematopoietic Stem Cell])/dt = reaction_1 - 2*reaction_2 - Kill_HSC
8. d(GMP)/dt = 2*reaction_5 - 2*reaction_8 + reaction_21 - 2*reaction_15 - Kill_GMP
9. d([Monocyte prog])/dt = 2*reaction_8 - 2*reaction_9 + reaction_22 - Kill_MonoP
10. d([B-lin])/dt = reaction_19 + reaction_32 - reaction_20
11. d([Gran-lin])/dt = 2*reaction_10 + reaction_28 - 2*reaction_31 - Kill_Gran
12. d([Gran-lin prog])/dt = -2*reaction_10 + 2*reaction_15 + reaction_4 - Kill_GranP
13. d([Prolif:Neutrophil-lin])/dt = 2*reaction_31 + reaction_16 - reaction_18
14. d([Quies:Neutrophil-lin])/dt = -reaction_23
15. d(totalDeadCells)/dt = reaction_7 + reaction_11 + reaction_17 + reaction_18 + reaction_20 + reaction_23 + Kill_EryI + Kill_MPP + Kill_HSC + Kill_MonoP + Kill_GMP + Kill_GranP + Kill_Gran + Kill_LymP

Events:

1. time>=0.01 = [Quies:Neutrophil-lin] = QF_Neutrophil*initNeutrophil
   [Prolif:Neutrophil-lin] = (1-QF_Neutrophil)*initNeutrophil

Reaction Rules

1. totalViableCells = [Neutrophil-lin]+[Lymphoid prog]+[Monocyte-lin]+[Erythroid-lin I]+[Erythroid-lin II]+[MK-lin]+[Gran-lin prog]+MPP+[Hematopoietic Stem Cell]+GMP+[Monocyte prog]+[B-lin]+[Gran-lin]
2. initNeutrophil = [Prolif:Neutrophil-lin]
3. [Neutrophil-lin] = [Prolif:Neutrophil-lin]+[Quies:Neutrophil-lin]
4. viability = totalViableCells/(totalViableCells+totalDeadCells)
5. kpro_MPP = kpro_MPP_0*(1-min(1,Emax_drug_MPP)*drug/(exp(log_EC50_MPP)*EC50_unit+drug))
6. kpro_HSC = kpro_HSC_0*(1-min(1,Emax_drug_HSC)*drug/(exp(log_EC50_HSC)*EC50_unit+drug))
7. kpro_GMP = kpro_GMP_0*(1-min(1,Emax_drug_GMP)*drug/(exp(log_EC50_GMP)*EC50_unit+drug))
8. kpro_ErythroidI = kpro_ErythroidI_0*(1-min(1,Emax_drug_ErythroidI)*drug/(exp(log_EC50_ErythroidI)*EC50_unit+drug))
9. kpro_ErythroidII = kpro_ErythroidII_0*(1-min(1,Emax_drug_ErythroidII)*drug/(exp(log_EC50_ErythroidII)*EC50_unit+drug))
10. kpro_MK = kpro_MK_0*(1-min(1,Emax_drug_MK)*drug/(exp(log_EC50_MK)*EC50_unit+drug))
11. kpro_MonoP = kpro_MonoP_0*(1-min(1,Emax_drug_MonoP)*drug/(exp(log_EC50_MonoP)*EC50_unit+drug))
12. kpro_Mono = kpro_Mono_0*(1-min(1,Emax_drug_Mono)*drug/(exp(log_EC50_Mono)*EC50_unit+drug))
13. kpro_GranP = kpro_GranP_0*(1-min(1,Emax_drug_GranP)*drug/(exp(log_EC50_GranP)*EC50_unit+drug))
14. kpro_Gran = kpro_Gran_0*(1-min(1,Emax_drug_Gran)*drug/(exp(log_EC50_Gran)*EC50_unit+drug))
15. kpro_Neut = kpro_Neut_0*(1-min(1,Emax_drug_Neut)*drug/(exp(log_EC50_Neut)*EC50_unit+drug))
16. kpro_B = kpro_B_0*(1-min(1,Emax_drug_B)*drug/(exp(log_EC50_B)*EC50_unit+drug))
17. Emax_cellkill_B = max(0,Emax_drug_B-1)*one_over_day
18. Emax_cellkill_ErythroidI = max(0,Emax_drug_ErythroidI-1)*one_over_day
19. Emax_cellkill_ErythroidII = max(0,Emax_drug_ErythroidII-1)*one_over_day
20. Emax_cellkill_GMP = max(0,Emax_drug_GMP-1)*one_over_day
21. Emax_cellkill_Gran = max(0,Emax_drug_Gran-1)*one_over_day
22. Emax_cellkill_GranP = max(0,Emax_drug_GranP-1)*one_over_day
23. Emax_cellkill_HSC = max(0,Emax_drug_HSC-1)*one_over_day
24. Emax_cellkill_LymP = max(0,Emax_drug_LymP-1)*one_over_day
25. Emax_cellkill_MK = max(0,Emax_drug_MK-1)*one_over_day
26. Emax_cellkill_Mono = max(0,Emax_drug_Mono-1)*one_over_day
27. Emax_cellkill_MonoP = max(0,Emax_drug_MonoP-1)*one_over_day
28. Emax_cellkill_MPP = max(0,Emax_drug_MPP-1)*one_over_day
29. Emax_cellkill_Neut = max(0,Emax_drug_Neut-1)*one_over_day

Compartments:

Name	Scope	Initial Value	Units
InVitro	Multilineage_InvitroHem	1.0	milliliter

Species - InVitro:

Name	Initial Value	Units
B-lin	55.595	molecule
drug	0.0	nanomole/liter
Erythroid-lin I	497.83	molecule
Erythroid-lin II	6.0643	molecule
GMP	754.32	molecule
Gran-lin	251.64	molecule
Gran-lin prog	34.342	molecule
Hematopoietic Stem Cell	275.67	molecule
Lymphoid prog	562.67	molecule
MK-lin	11.616	molecule
Monocyte prog	86.607	molecule
Monocyte-lin	32.419	molecule
MPP	487.71	molecule
Prolif:Neutrophil-lin	154.07	molecule
Quies:Neutrophil-lin	0.0	molecule
totalDeadCells	875.55	molecule
totalViableCells	3210.5533	molecule

Parameters (Model Scoped):

Name	Initial Value	Units
EC50_unit	1.0	nanomole/liter
Emax_cellkill_B	0.0	1/day
Emax_cellkill_ErythroidI	0.0	1/day
Emax_cellkill_ErythroidII	0.0	1/day
Emax_cellkill_GMP	0.0	1/day
Emax_cellkill_Gran	0.0	1/day
Emax_cellkill_GranP	0.0	1/day
Emax_cellkill_HSC	0.0	1/day
Emax_cellkill_LymP	0.0	1/day
Emax_cellkill_MK	0.0	1/day
Emax_cellkill_Mono	0.0	1/day
Emax_cellkill_MonoP	0.0	1/day
Emax_cellkill_MPP	0.0	1/day
Emax_cellkill_Neut	0.0	1/day
Emax_drug_B	0.0	dimensionless
Emax_drug_ErythroidI	0.0	dimensionless
Emax_drug_ErythroidII	0.0	dimensionless
Emax_drug_GMP	0.0	dimensionless
Emax_drug_Gran	0.0	dimensionless

Parameters (Model Scoped):

Name	Initial Value	Units
Emax_drug_GranP	0.0	dimensionless
Emax_drug_HSC	0.0	dimensionless
Emax_drug_LymP	0.0	dimensionless
Emax_drug_MK	0.0	dimensionless
Emax_drug_Mono	0.0	dimensionless
Emax_drug_MonoP	0.0	dimensionless
Emax_drug_MPP	0.0	dimensionless
Emax_drug_Neut	0.0	dimensionless
initNeutrophil	154.07	molecule
kbranch_Erythroid	0.67317	dimensionless
kbranch_GMP	0.30481	dimensionless
kbranch_MK	0.021653	dimensionless
kbranch_Mono	0.46395	dimensionless
kDeath	0.45887	1/day
kdiff_lym	0.076318	1/day
kpro_B	0.84171	1/day
kpro_B_0	0.84171	1/day
kpro_ErythroidI	0.0077462	1/day
kpro_ErythroidI_0	0.0077462	1/day
kpro_ErythroidII	1.9612	1/day
kpro_ErythroidII_0	1.9612	1/day
kpro_GMP	0.27694	1/day
kpro_GMP_0	0.27694	1/day
kpro_Gran	1.1726E-4	1/day
kpro_Gran_0	1.1726E-4	1/day
kpro_GranP	5.7012	1/day
kpro_GranP_0	5.7012	1/day
kpro_HSC	5.2597	1/day
kpro_HSC_0	5.2597	1/day
kpro_MK	1.072	1/day
kpro_MK_0	1.072	1/day
kpro_Mono	1.3532	1/day
kpro_Mono_0	1.3532	1/day
kpro_MonoP	0.15714	1/day
kpro_MonoP_0	0.15714	1/day
kpro_MPP	5.4285	1/day
kpro_MPP_0	5.4285	1/day
kpro_Neut	2.6679	1/day
kpro_Neut_0	2.6679	1/day
log_EC50_B	1.0	dimensionless
log_EC50_ErythroidI	1.0	dimensionless
log_EC50_ErythroidII	1.0	dimensionless
log_EC50_GMP	1.0	dimensionless
log_EC50_Gran	1.0	dimensionless

Parameters (Model Scoped):

Name	Initial Value	Units
log_EC50_GranP	1.0	dimensionless
log_EC50_HSC	1.0	dimensionless
log_EC50_LymP	1.0	dimensionless
log_EC50_MK	1.0	dimensionless
log_EC50_Mono	1.0	dimensionless
log_EC50_MonoP	1.0	dimensionless
log_EC50_MPP	1.0	dimensionless
log_EC50_Neut	1.0	dimensionless
Neutrophil-lin	154.07	molecule
one_cell	1.0	molecule
one_cell_per_ml	1.0	molecule/milliliter
one_over_day	1.0	1/day
QF_Neutrophil	0.99974	dimensionless
renewal_ErythroidI	0.33835	dimensionless
renewal_GMP	0.47546	dimensionless
renewal_Gran	0.36766	dimensionless
renewal_GranP	0.49822	dimensionless
renewal_HSC	0.54755	dimensionless
renewal_Mono	0.26505	dimensionless
renewal_MonoP	0.41977	dimensionless
renewal_MPP	0.21625	dimensionless
viability	0.785724947286575	dimensionless
