# Supplementary material for: An in vitro quantitative systems pharmacology approach for deconvolving mechanisms of drug-induced, multilineage cytopenias
Source: PLoS Comput Biol. 2020 Jul 23;16(7):e1007620. doi: 10.1371/journal.pcbi.1007620 (PMC7402526; doi:10.1371/journal.pcbi.1007620)
Supplement: S2 Text — (PDF) [file pcbi.1007620.s005.pdf]

We employed the Matlab function `sbioparameterci` [1] to address the question of identifiability of parameters via profile likelihood. In particular, we examined the 90% confidence level of parameters, corresponding to ‘`Alpha`’=0.1. We chose the algorithm settings as '`Tolerance`'=0.1 and '`MaxStepSize`'=0.5.

As part of our estimation process, we have provided upper and lower bounds based on biological plausibility for all systems parameters, as well as an inequality constraint for a set of 3 branching parameters (‘kbranch_Erythroid’+’kbranch_MK’+’kbranch_GMP’≤1) based on a conservation requirement. Note that the current profile likelihood algorithm [1] can only handle lower and upper bounds, but cannot account for additional linear or nonlinear constraints placed on the parameters to be identified. Hence, in our identifiability analysis we have retained the two larger branching parameters ‘kbranch_Erythroid’ and ‘kbranch_GMP’ within the equality constraint, but left out the smallest parameter ‘kbranch_MK’. In addition, we have fixed the values of renewal fractions and left them out of the analysis; we discuss this aspect further below.

The following table shows the list of model parameters we have used in profile likelihood analysis and the obtained outcomes. The results indicate that other than a few parameters that are constrained by *a priori* upper and lower bounds (notably ‘kdiff_lym’ and ‘kpro_Gran' reaching the lower positivity constraint; 'kpro_HSC' and ‘kpro_GranP’ reaching the upper bounds), we have reasonable ranges of confidence interval for the systems parameters.

Name	Estimate	Bounds		Confidence Interval	
'kdiff_lym'	0.065533	0	3	0.065533	0.1181
'kbranch_GMP'	0.30412	0.001	1	0.22834	0.513
'kbranch_Mono'	0.46724	0.001	1	0.36207	0.574
'kbranch_Erythroid'	0.69957	0.001	1	0.55883	0.96777
'kpro_HSC'	5.3706	0	5.7708	4.9181	5.7708
'kpro_MPP'	5.7646	0	5.7708	3.4032	5.7646
'kpro_GMP'	0.27845	0	5.7708	0.20884	0.397
'kpro_MK'	1.1314	0	5.7708	0.9	1.523
'kpro_ErythroidI'	0.007485	0	5.7708	0.0075	0.0203
'kpro_ErythroidII'	2.015	0	5.7708	1.6591	2.4086
'kpro_Gran'	6.89e-07	0	5.7708	6.89e-07	0.002
'kpro_Neut'	2.0684	0	5.7708	1.7335	2.4765
'kpro_Monn'	1.471	0	5.7708	0.72074	1.9975
'kpro_MonoP'	0.12005	0	5.7708	0	0.66317
'kpro_GranP'	5.7703	0	5.7708	4.7147	5.7703
'kpro_B'	0.9254	0	5.7708	0.66612	1.2835
'QF_Neutrophil'	0.99017	0	1	0.97661	0.99
'kDeath'	0.52059	0	2	0.30448	0.85992

Regarding the renewal fraction parameters, we have bounds based on biological considerations. Due to the underlying assumption that HSCs are self-replenishing, we have a

bound constraint for the renewal fraction of HSC (i.e., 1/2 ≤ 'renewal_HSC' ≤ 1). In addition, assuming that the remaining cell populations do not self-replenish, the remaining renewal fraction parameters ('renewal_MPP', 'renewal_GMP', 'renewal_ErythroidI', 'renewal_Gran', 'renewal_Mono', 'renewal_MonoP', 'renewal_GranP') are constrained to lie between 0 and 1/2. Under further assumptions, additional equality constraints can be placed on the renewal parameters. Prior analysis has shown the key role that renewal fraction parameters play in the amplification of cell numbers as they differentiate along a given lineage [2]. In particular, if the system reaches steady-state the cell numbers of each lineage in relation to each other place constraints on the renewal fractions. While the *in-vitro* assay is not a system at steady-state, the cell numbers in the *in-vivo* situation from healthy donors are at an equilibrium. However, the proliferation kinetics is expected to be different between *in-vitro* and *in-vivo* scenarios. Relating *in-vivo* to *in-vitro* hematopoiesis is an area for further work.

[1] https://www.mathworks.com/help/simbio/ref/sbioparameterci.html

[2] Stiehl T, Marciniak-Czochra A. Stem cell self-renewal in regeneration and cancer: insights from mathematical modeling. Current Opinion in Systems Biology. 2017 Oct 1;5:112-20.
