# Supplementary material for: An in vitro quantitative systems pharmacology approach for deconvolving mechanisms of drug-induced, multilineage cytopenias
Source: PLoS Comput Biol. 2020 Jul 23;16(7):e1007620. doi: 10.1371/journal.pcbi.1007620 (PMC7402526; doi:10.1371/journal.pcbi.1007620)
Supplement: S3 Text — (PDF) [file pcbi.1007620.s006.pdf]

**Parameter Name in paper and figures**	**Example parameter**	**Parameter name in MATLAB code**
Renewal parameter, ρ	ρ_MPP	renewal_MPP, renewal_GMP, renewal_MonoP, renewal_GranP, renewal_ErythroidI, renewal_Gran
Proliferation parameter, κ	κ_MPP	kpro_HSC, kpro_MPP, kpro_GMP, kpro_MonoP, kpro_GranP, kpro_ErythroidI, kpro_MK, kpro_ErythroidII, kpro_Gran, kpro_Mono, kpro_B, kpro_Neut
Branching parameter, β	β_GMP	kbranch_GMP, kbranch_Erythroid, kbranch_MK, kbranch_Mono
Death rate, δ	δ	kDeath
Total drug effect, $Emax_T$	$Emax_T$GMP	Emax_drug_ErythroidII, Emax_drug_MK, Emax_drug_Mono, Emax_drug_Neut, Emax_drug_B, Emax_drug_ErythroidI, Emax_drug_MPP, Emax_drug_HSC, Emax_drug_MonoP, Emax_drug_GMP, Emax_drug_GranP, Emax_drug_Gran, Emax_drug_LymP
Cell-killing drug effect, EmaxCK	EmaxCK_GMP	Emax_cellkill_ErythroidII, Emax_cellkill_MK, Emax_cellkill_Mono, Emax_cellkill_Neut, Emax_cellkill_B, Emax_cellkill_ErythroidI, Emax_cellkill_MPP, Emax_cellkill_HSC, Emax_cellkill_MonoP, Emax_cellkill_GMP, Emax_cellkill_GranP, Emax_cellkill_Gran, Emax_cellkill_LymP
EC50, measured in the model as the log(EC50)	EC50_GMP	log_EC50_ErythroidII, log_EC50_MK, log_EC50_Mono, log_EC50_Neut, log_EC50_B, log_EC50_ErythroidI, log_EC50_MPP, log_EC50_HSC, log_EC50_MonoP, log_EC50_GMP, log_EC50_GranP, log_EC50_Gran, log_EC50_LymP
