# Supplementary figures and images for: An in vitro quantitative systems pharmacology approach for deconvolving mechanisms of drug-induced, multilineage cytopenias
Source: PLoS Comput Biol. 2020 Jul 23;16(7):e1007620. doi: 10.1371/journal.pcbi.1007620 (PMC7402526; doi:10.1371/journal.pcbi.1007620)

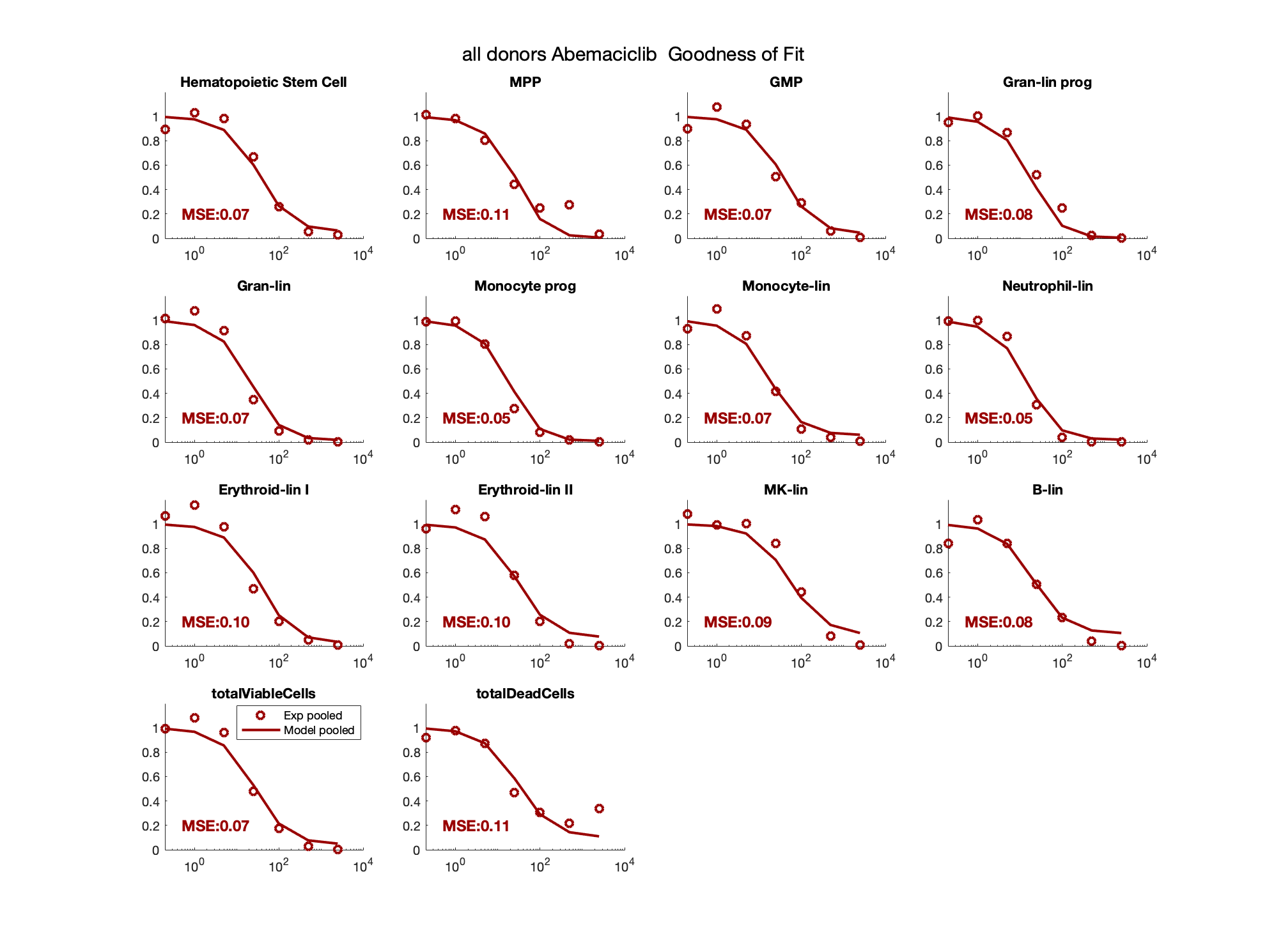

Supplement: S1 Fig — For each cell type and total live and viable cells, normalized cell counts from experimental data (open circles) and simulated results (solid line) are plotted against concentration (nM). Additionally, each plot includes the mean squared error of the difference between experimental and data plotted with the estimated EmaxT effects. (PNG) [file pcbi.1007620.s007.png]

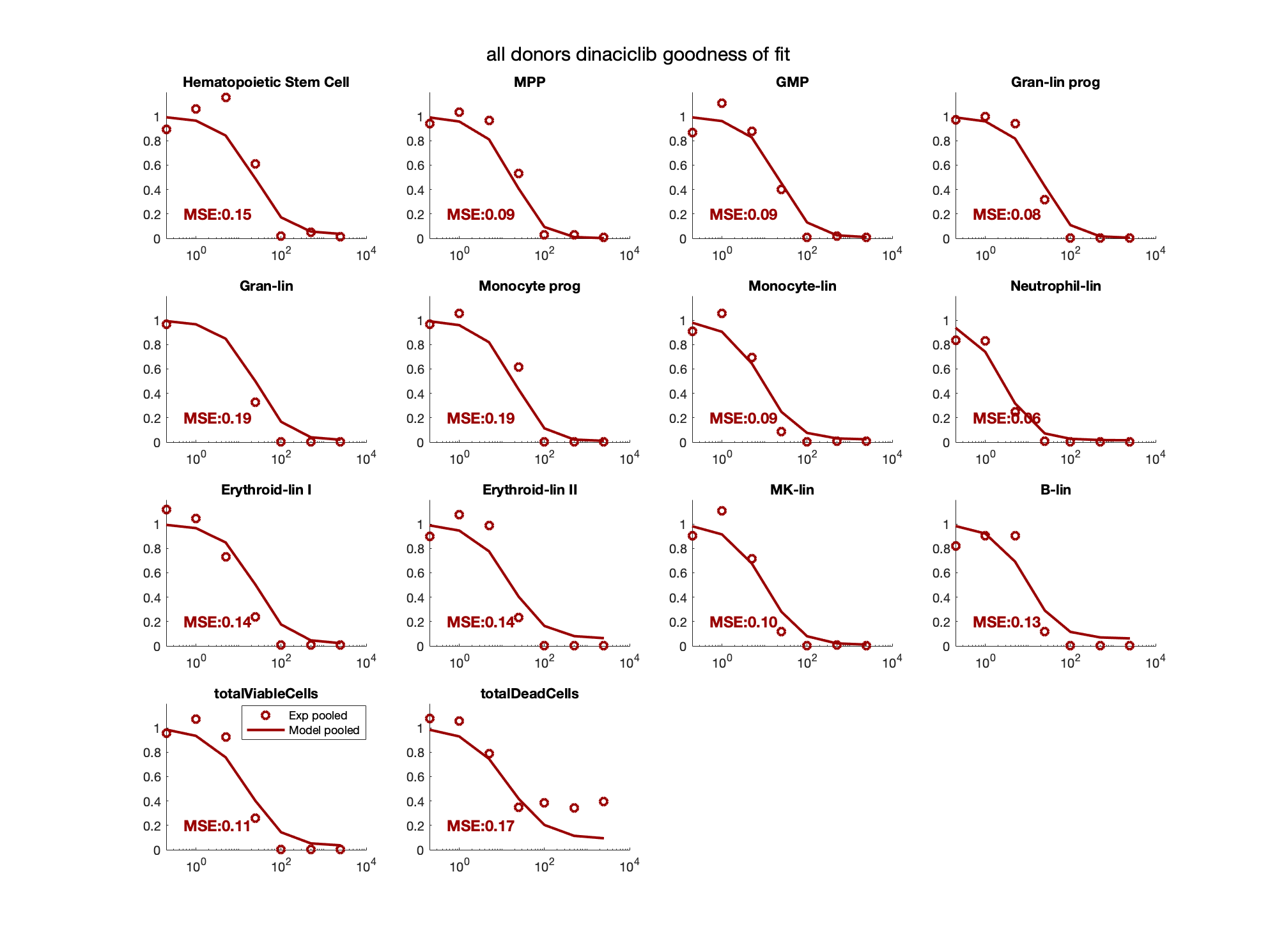

Supplement: S2 Fig — For each cell type and total live and viable cells, normalized cell counts from experimental data (open circles) and simulated results (solid line) are plotted against concentration (nM). Additionally, each plot includes the mean squared error of the difference between experimental and data plotted with the estimated EmaxT effects. (PNG) [file pcbi.1007620.s008.png]

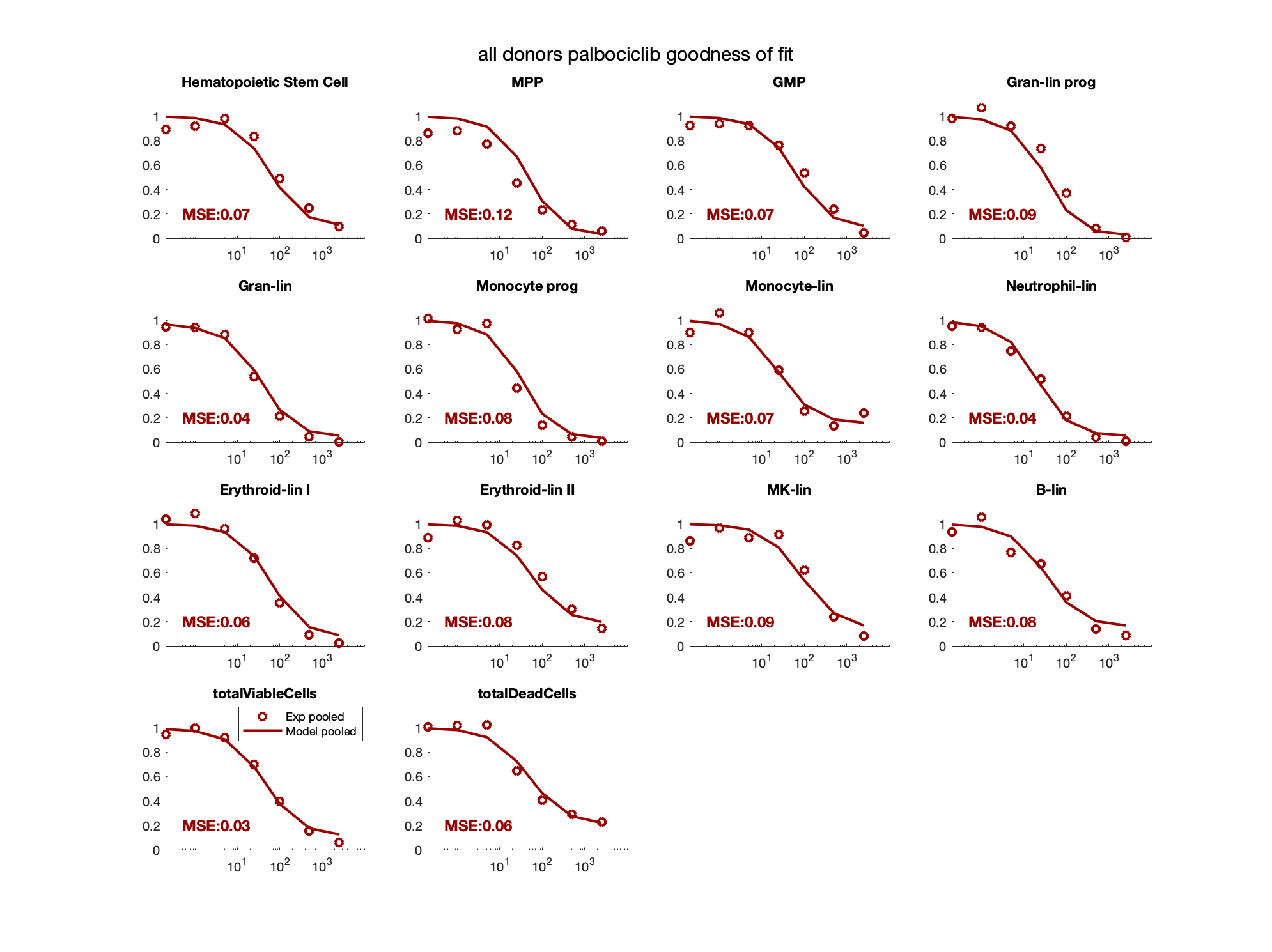

Supplement: S3 Fig — For each cell type and total live and viable cells, normalized cell counts from experimental data (open circles) and simulated results (solid line) are plotted against concentration (nM). Additionally, each plot includes the mean squared error of the difference between experimental and data plotted with the estimated EmaxT effects. (PNG) [file pcbi.1007620.s009.png]

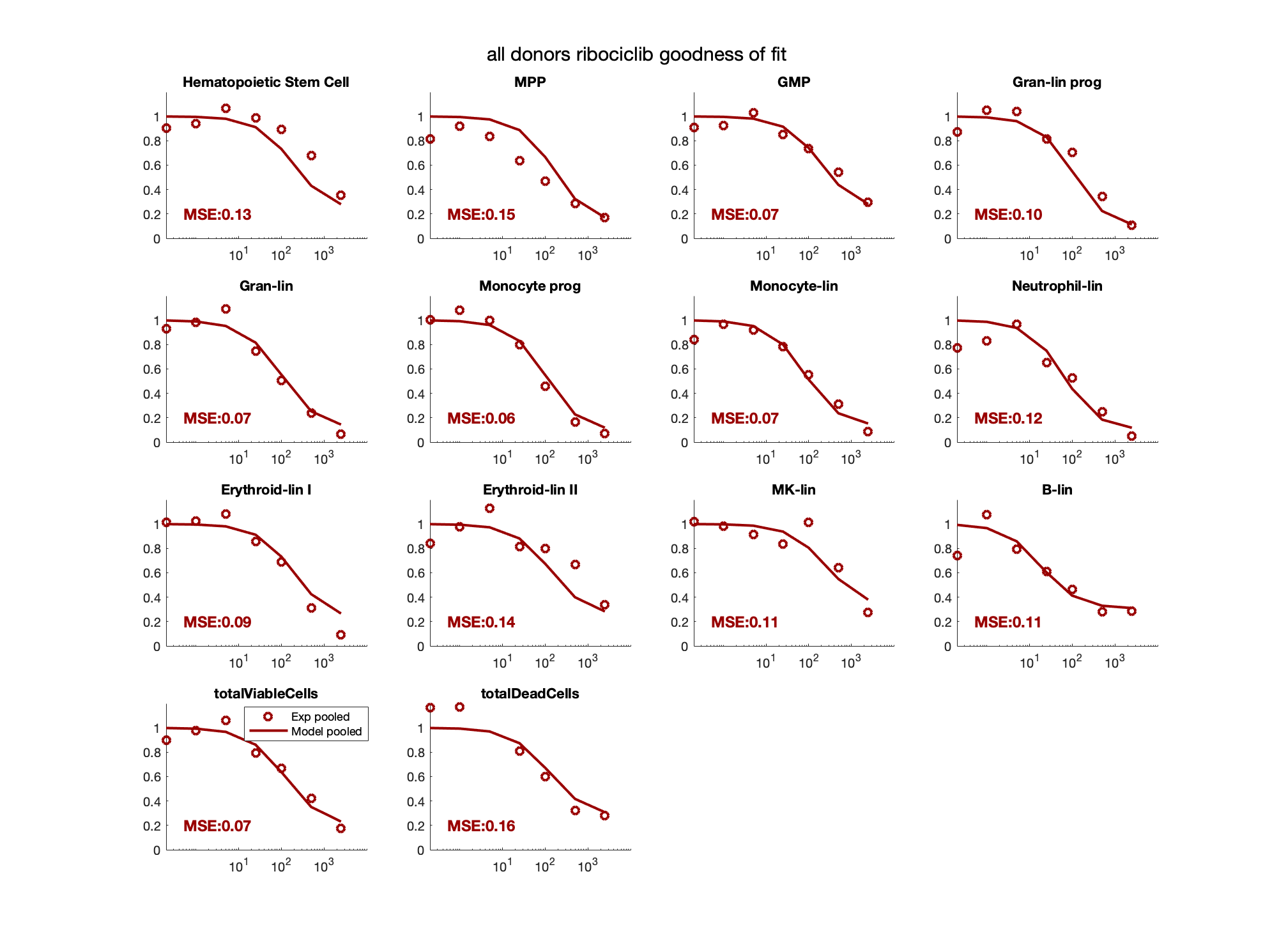

Supplement: S4 Fig — For each cell type and total live and viable cells, normalized cell counts from experimental data (open circles) and simulated results (solid line) are plotted against concentration (nM). Additionally, each plot includes the mean squared error of the difference between experimental and data plotted with the estimated EmaxT effects. (PNG) [file pcbi.1007620.s010.png]

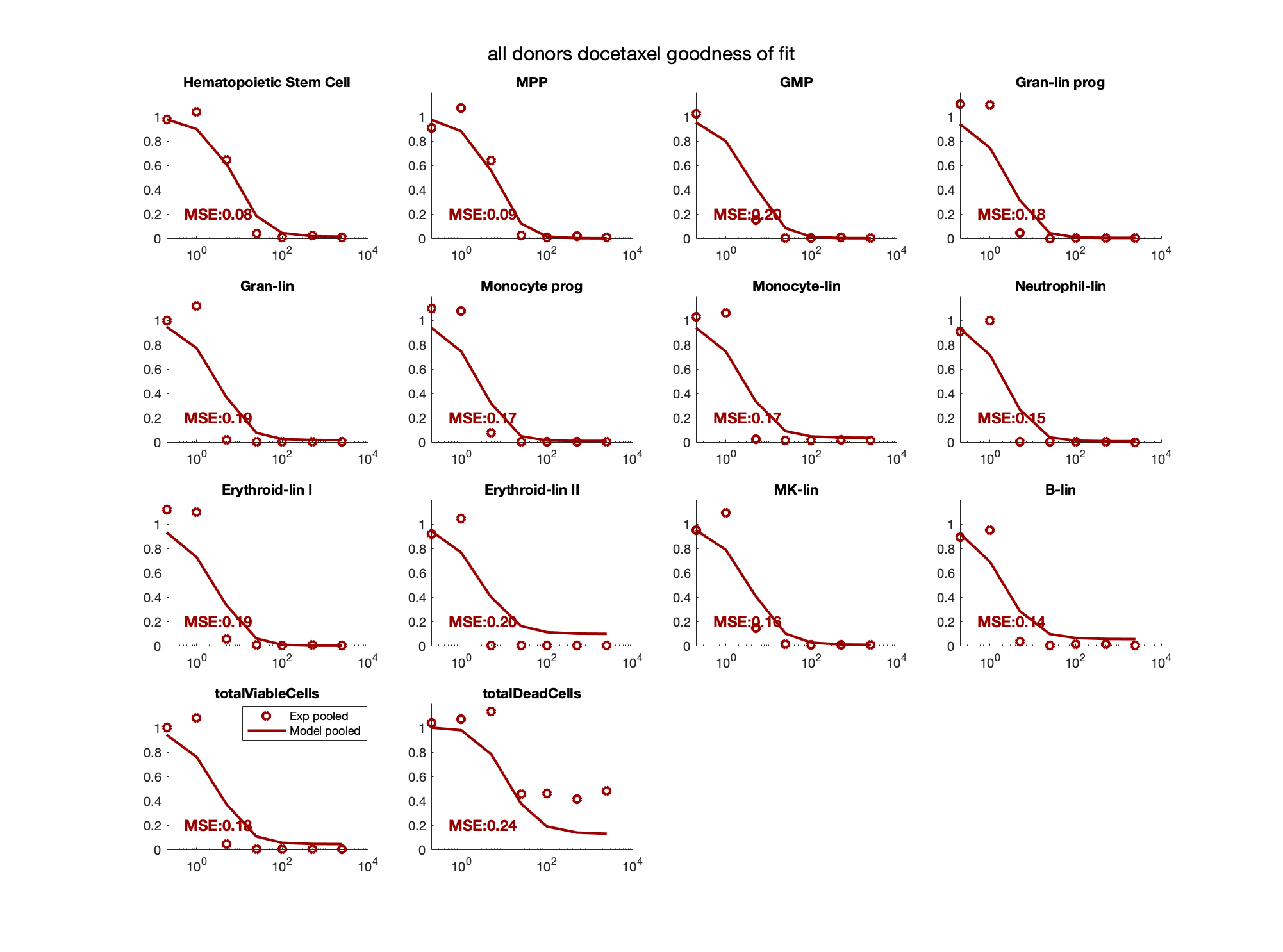

Supplement: S5 Fig — For each cell type and total live and viable cells, normalized cell counts from experimental data (open circles) and simulated results (solid line) are plotted against concentration (nM). Additionally, each plot includes the mean squared error of the difference between experimental and data plotted with the estimated EmaxT effects. (PNG) [file pcbi.1007620.s011.png]

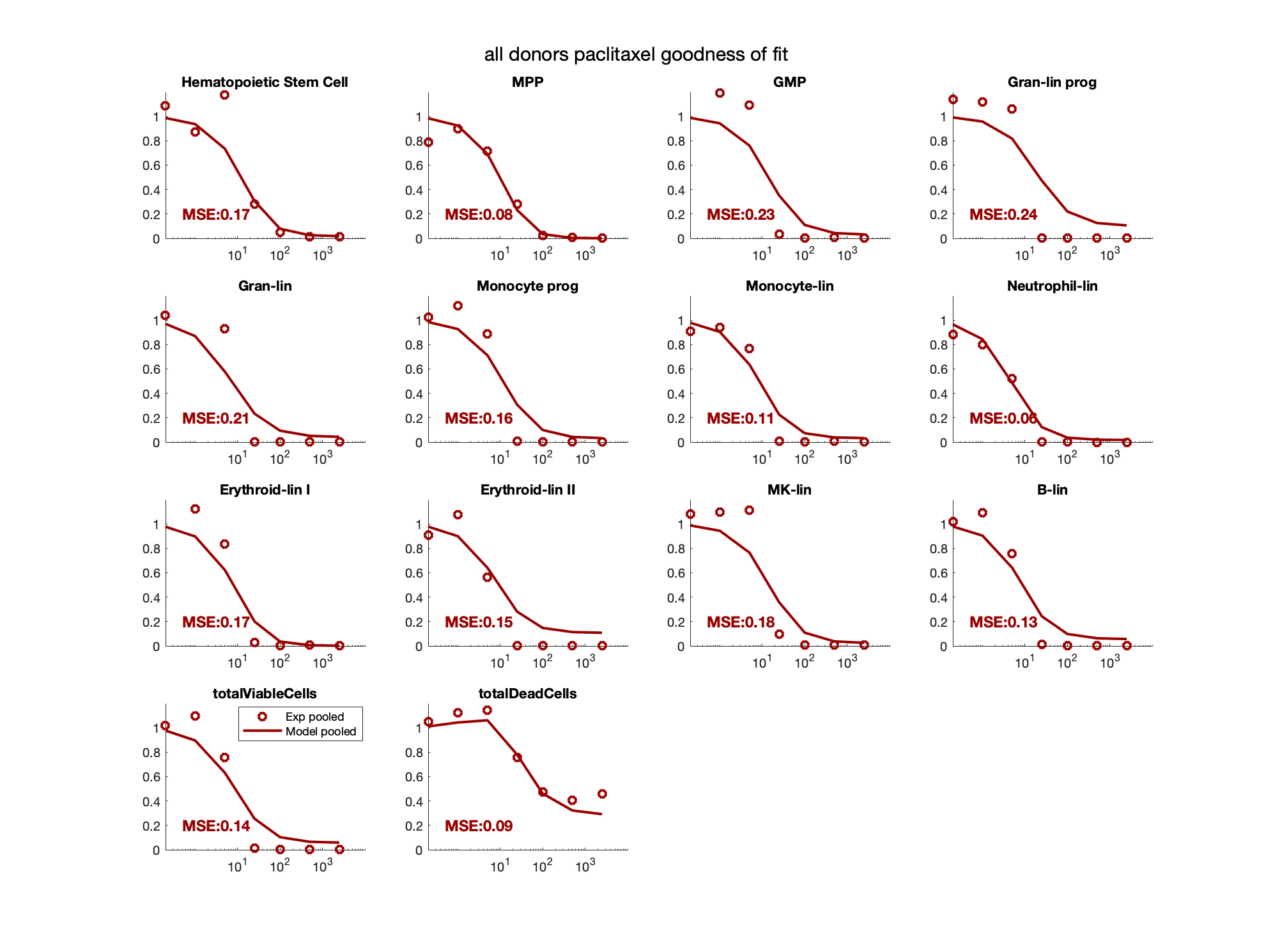

Supplement: S6 Fig — For each cell type and total live and viable cells, normalized cell counts from experimental data (open circles) and simulated results (solid line) are plotted against concentration (nM). Additionally, each plot includes the mean squared error of the difference between experimental and data plotted with the estimated EmaxT effects. (PNG) [file pcbi.1007620.s012.png]

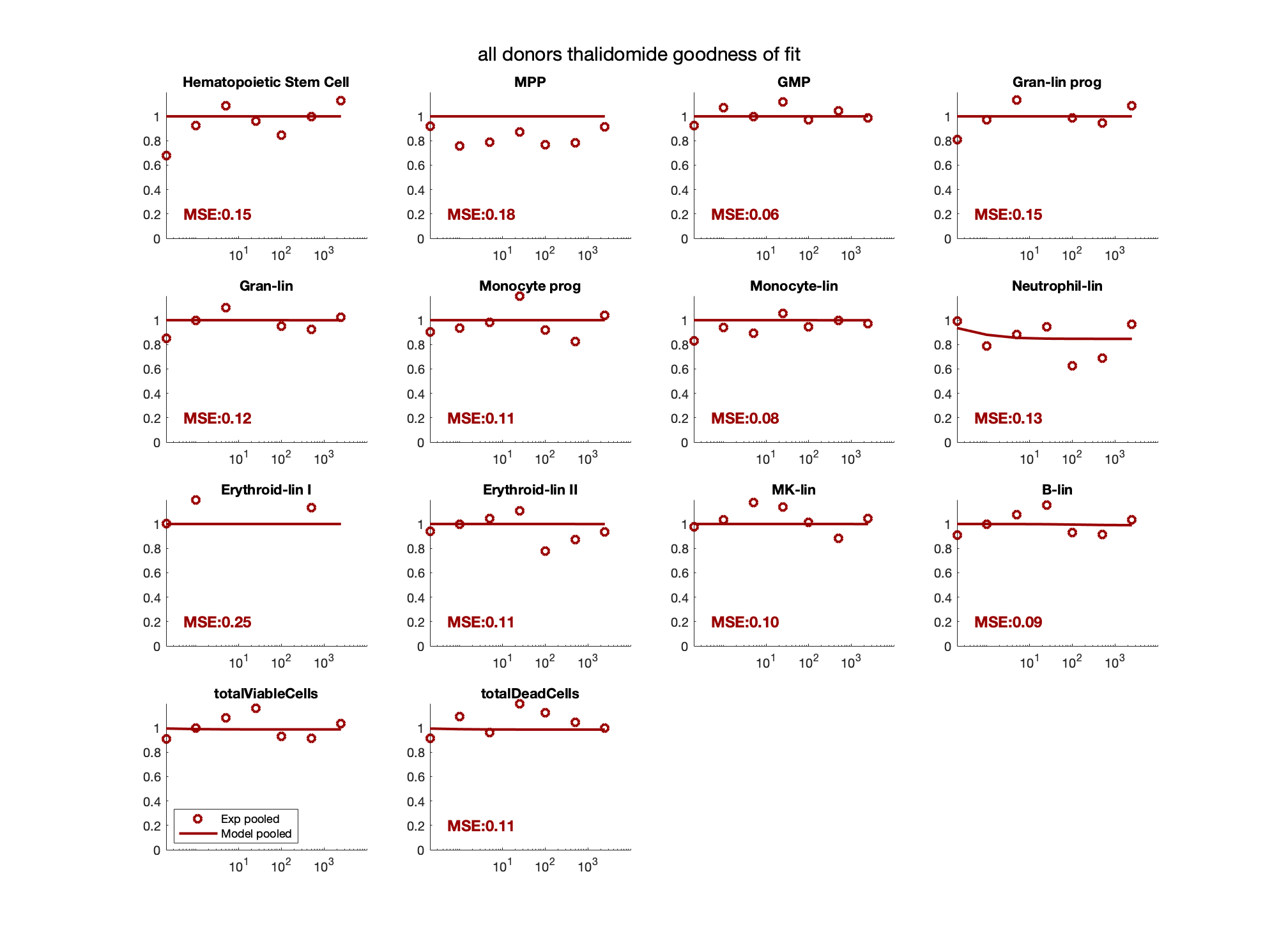

Supplement: S7 Fig — For each cell type and total live and viable cells, normalized cell counts from experimental data (open circles) and simulated results (solid line) are plotted against concentration (nM). Additionally, each plot includes the mean squared error of the difference between experimental and data plotted with the estimated EmaxT effects. (PNG) [file pcbi.1007620.s013.png]

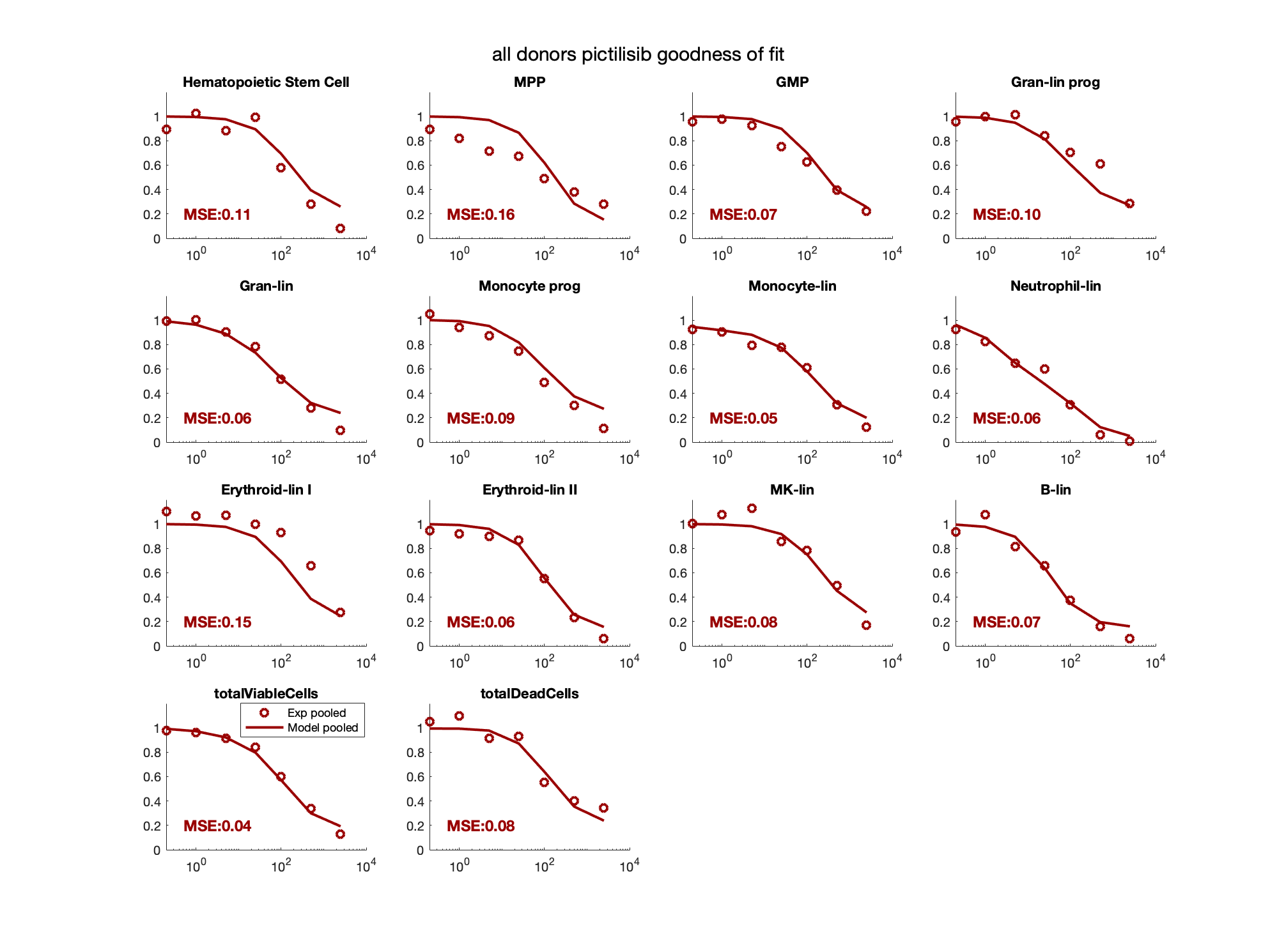

Supplement: S8 Fig — For each cell type and total live and viable cells, normalized cell counts from experimental data (open circles) and simulated results (solid line) are plotted against concentration (nM). Additionally, each plot includes the mean squared error of the difference between experimental and data plotted with the estimated EmaxT effects. (PNG) [file pcbi.1007620.s014.png]

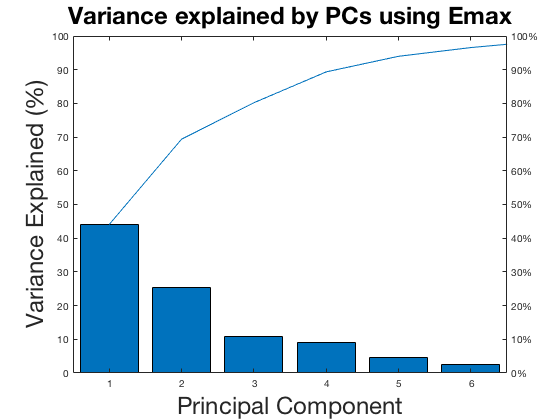

Supplement: S9 Fig — The percent variance(left axis) and cumulative variance (right axis) are plotted against the top six components (x-axis) for PCA analysis of EmaxT values. (PNG) [file pcbi.1007620.s015.png]

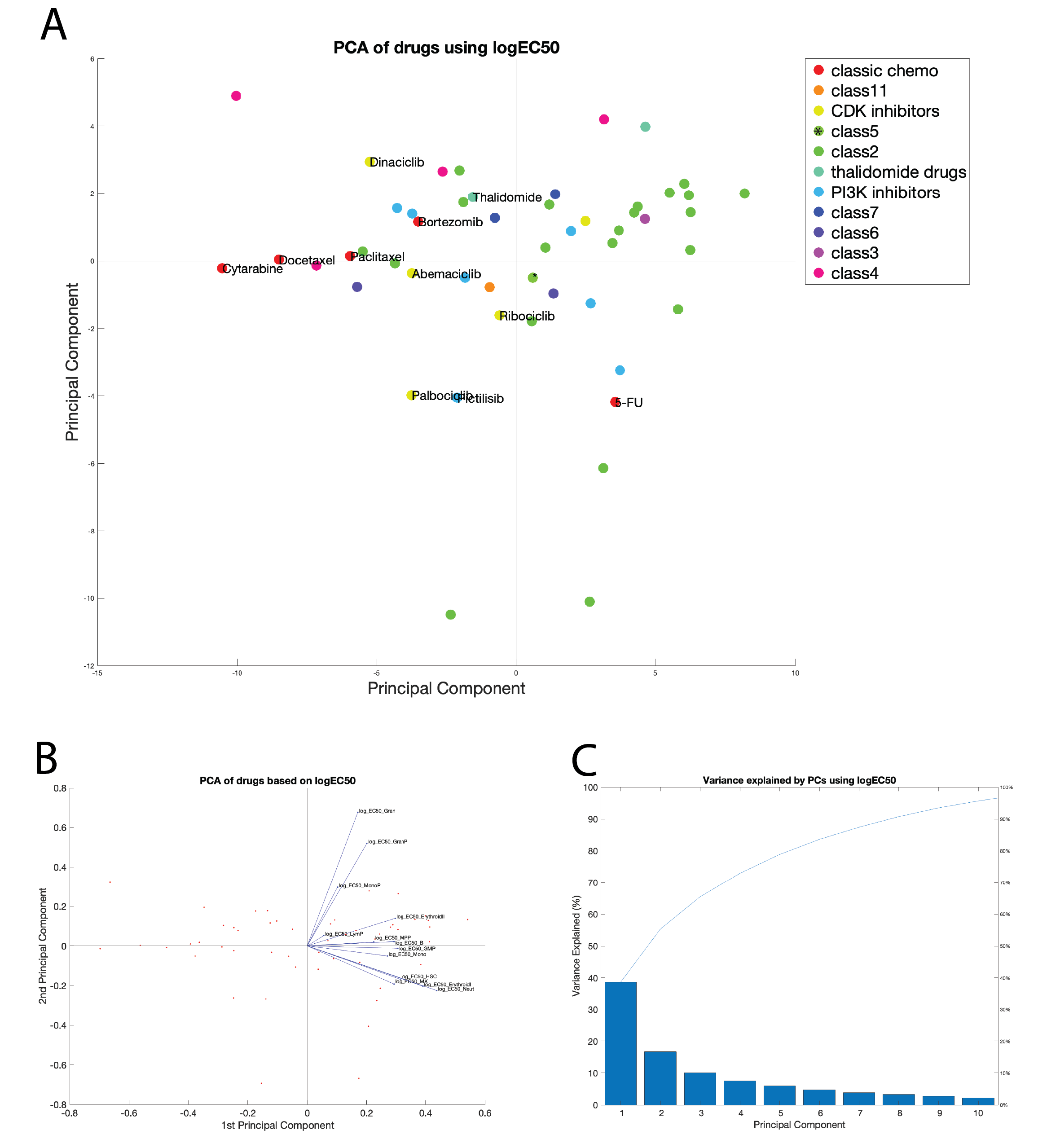

Supplement: S10 Fig — The 51 compounds are plotted in PCA space (A). Marker color corresponds to drug class. Drugs and variables contributing to the top two components are plotted in PCA space (B). Note: in figure A there is only one drug in class 5 and it is marked with an * to distinguish this compound from the remaining class 2 drugs. Variance explained by each principal component is plotted in (C). (PNG) [file pcbi.1007620.s016.png]

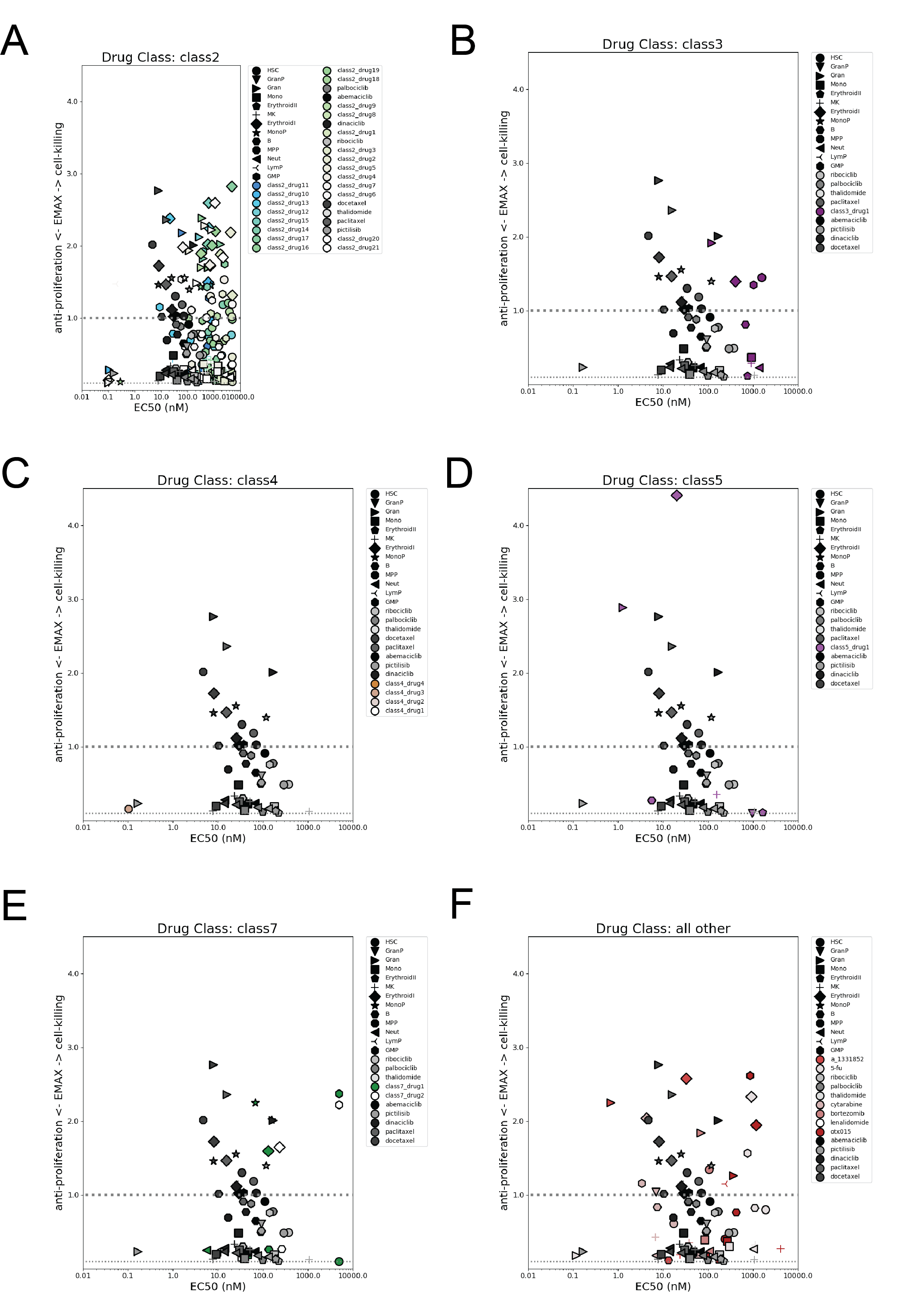

Supplement: S11 Fig — Emax parameters per cell type are plotted against the EC50 values for each cell type. Marker shape represents cell type and marker shading represents either the sample set (black gradient, all figures) or drugs in class 2 (blues, A), class 3 (purples, B), class 4 (browns, C), class 5 (orchids, D), class 7 (green, E), or all other drugs (reds, F). The dashed line represents where Emax = 1.0. (PNG) [file pcbi.1007620.s017.png]
